# Artificial Intelligence in Dermatology Image Analysis: Current Developments and Future Trends

**DOI:** 10.3390/jcm11226826

**Published:** 2022-11-18

**Authors:** Zhouxiao Li, Konstantin Christoph Koban, Thilo Ludwig Schenck, Riccardo Enzo Giunta, Qingfeng Li, Yangbai Sun

**Affiliations:** 1 Department of Plastic and Reconstructive Surgery, Shanghai 9th People’s Hospital, Shanghai Jiao Tong University School of Medicine, Shanghai 200023, China; 2 Division of Hand, Plastic and Aesthetic Surgery, University Hospital, LMU Munich, 80339 Munich, Germany

**Keywords:** deep learning, pattern recognition, dermatology, skin cancer, intelligent diagnosis, 3D imaging

## Abstract

Background: Thanks to the rapid development of computer-based systems and deep-learning-based algorithms, artificial intelligence (AI) has long been integrated into the healthcare field. AI is also particularly helpful in image recognition, surgical assistance and basic research. Due to the unique nature of dermatology, AI-aided dermatological diagnosis based on image recognition has become a modern focus and future trend. Key scientific concepts of review: The use of 3D imaging systems allows clinicians to screen and label skin pigmented lesions and distributed disorders, which can provide an objective assessment and image documentation of lesion sites. Dermatoscopes combined with intelligent software help the dermatologist to easily correlate each close-up image with the corresponding marked lesion in the 3D body map. In addition, AI in the field of prosthetics can assist in the rehabilitation of patients and help to restore limb function after amputation in patients with skin tumors. The aim of the study: For the benefit of patients, dermatologists have an obligation to explore the opportunities, risks and limitations of AI applications. This study focuses on the application of emerging AI in dermatology to aid clinical diagnosis and treatment, analyzes the current state of the field and summarizes its future trends and prospects so as to help dermatologists realize the impact of new technological innovations on traditional practices so that they can embrace and use AI-based medical approaches more quickly.

## 1. Introduction

Long considered futuristic, artificial intelligence has now substantially improved our quality of life through the instrumentalization of machines and robots in industry, autonomous driving and the widespread use of smartphones [1]. Recent years have also seen significant improvements in the productivity, accuracy and efficiency of AI-optimized workflows in the healthcare sector. Deep learning and convolutional cloud neural-network-based algorithms can greatly improve the efficiency of image classification, object detection, segmentation, registration and other tasks [2]. In those areas of medicine that rely on imaging data, AI medical image recognition and analysis is greatly beneficial for high-speed, high-precision diagnosis alongside professional evaluation, especially in the dermatology area. The massive learning capacity of AI allows it to recognize subtle differences in lesion features such as size, texture and shades, and far surpasses that of humans [3,4,5].

The trend towards digitization and technology has been happening in the dermatological field for a while [6,7,8,9]. As a morphological feature-dependent discipline, dermatology plays a groundbreaking role in the utilization of AI for diagnostics and assessment [10]. The burgeoning technology offer a precious and valuable chance for dermatologists. They should comprehensively know of the utilization and limitation of this novel tool, and propel its safe and effective implementation [11]. Regarding diagnosis, AI’s ability to learn skin lesions’ features far exceeds that of humans, allowing it to quantify lesion features and make judgements to assist in the discovery and analysis of lesions, improving the accuracy and efficiency of clinicians’ diagnosis [12]. In terms of treatment, AI can select the best treatment for the patient and predict the number of treatments required and the efficacy of the treatment for patients with skin diseases [13,14]. AI-based surgical robotic systems can also help to reduce manpower consumption, eliminate human fatigue and potential errors and significantly reduce surgery times, as well as improve the surgical treatment [15,16]. For these above reasons, we explain the definition of AI and the core ultimate principles and technology to help dermatologists and dermatologic surgeons understand how AI works and how these procedures are accomplished. We outlined the relevant developments and applications of AI in dermatology and discussed the attitudes of different populations towards AI.

Although there are several reviews summarizing the application of AI in dermatology, they mainly focused on the implementation of AI for binary-classification of skin disease and were arranged as different sections, one for each disease. Thus, the problem addressed in each is mostly a binary classification of present/absent rather than considering the multi-class problem faced in real clinical scenarios where the patient comes to the doctor with any of them. In addition, the metadata representing information such as site, age and sex are not included in these studies, even though such information is collected by doctors in their examination of patients and is included in the diagnostic decision for doctors. It is therefore unrealistic to compare the performance of doctors with AI systems in this context [17,18,19]. The papers of the winners of the International Skin Imaging Collaboration (ISIC) annual competitions in the past several years (2016 to 2020), which represent the benchmark for different research groups working in this area, were included in the literature surveyed in this work. The ISIC challenges consider AI systems that can identify the presence of many different pathologies and make the metadata available for the labeled cases, therefore allowing for a more realistic comparison between AI systems and clinical scenarios [20,21,22,23]. In addition, former reviews concentrated more on the mechanism and theory of an AI computer-aided diagnosis (CAD) system without a summarization of existing AI-CAD systems on the market. This may prove beneficial for dermatologists to better understand AI and learn its mechanisms in skin lesion image recognition, but it is of limited help in guiding them to apply AI in specific clinical practice.

To fill these gaps and to benefit more grassroots dermatologists than just researchers, in our current review:

We comprehensively summarize the birth and development of AI and focus on emerging AI-CAD applications in the dermatosis field, not only for binary classification but also for multiple classification. **Firstly,** we focus on emerging AI applications in aided clinical diagnoses and treatment, summarizing the development of artificial intelligence in the dermatosis field and providing a novel perspective for dermatological studies. **Secondly,** we describe not only the principle and mechanism of the AI system but also introduced the current AI CAD systems and products in the dermatology field on the market thoroughly, which provides detailed guide and perspectives for normal clinical practice. **Thirdly,** based on clinical practice, we have comprehensively analyzed the attitudes of healthcare workers and patients towards artificial intelligence. **Fourthly,** we make reasonable predictions and future trends about the use of AI in dermatology in the context of a domestic and international government policy document. **Lastly,** we objectively evaluated the potential and limitation of its application, along with the underlying ethical issues.

We hope that this all-round survey will allow more dermatologists to have a deeper and more intuitive understanding of AI-based diagnostic tools and medical means so that more dermatology patients can benefit from these emerging healthcare models.

## 2. Overview of AI

AI can be divided into two parts: “artificial” and “intelligence”. “Artificial “ means designed, created and manufactured by humans. The definition of intelligence remains controversial: it is widely accepted that the only intelligence is human intelligence, but our understanding of human intelligence is still limited [24]. Below, we will give a brief overview from the following three aspects.

### 2.1. What Is AI?

AI refers to the intelligence manifested by machines made by humans. It is used to describe machines (or computers) that mimic the “cognitive” functions associated with human thought, such as “learning” and “problem solving” [25]. Intelligent agents are systems which can observe their surroundings and adopt action to reach their targets directly [5], learn from them and use that knowledge to achieve specific goals and tasks with flexibility [26,27].

### 2.2. The History of AI and Its Development Path in Medicine

**Figure 1:**
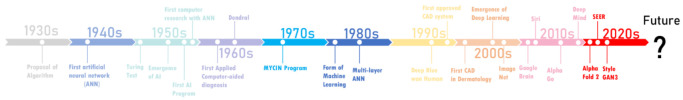
Flow chart illustrating the literature search and study selection.

In 1936, Alan Turing published a paper expounding on “Entscheidungsproblem” and proposed “effective calculability” for solving the problem. They laid the foundation of computational models called algorithms [28]. In 1943, the first artificial neural network made of electrical circuits was modeled to simulate brain neuronal interactions [29]. The concept of AI was born in 1956 at Dartmouth College [30]. Three years later, the first computer research using an ANN was completed through models called “ADALINE” and “MADALINE” [31]. In 1963, the computer-aid-diagnosis was firstly applied in the analysis of pulmonary nodules detected in chest radiographs [32]. Fifteen years after the birth of AI, scientists discovered its relevance in bioscience, most evidently in the Dendral experiments [33]. However, technology limited the depth of AI’s application in medicine until 1998, when the first mammography CAD system was approved by U.S.FDA [34]. Soon after, CAD was brought into dermatology. The MelaFind^TM^ multispectral digital dermoscopy system uses the same feature-based classifiers to discriminate the malignant melanoma from benign pigmented skin lesions [35]. After the 2010s, a subfield of machine learning called deep learning has emerged. Deep learning allows computers to learn certain features by themselves from large datasets without explicit programming [36]. The application of AI in medicine and dermatology has been growing exponentially into the 2020s. A pioneering work developed by DeepMind successfully predicted the 3D structure of proteins, the basic molecules of life [37]. The creation of a more powerful computer vision model, SEER, and a new generation of generative adversarial networks (Style GAN3) have provided more powerful tools for AI to learn from image sets, contributing to more robust dermatology AI CAD systems in future [38,39]. In Figure 1, more historical details are presented as a timeline.

### 2.3. Relevant Concept of AI in Dermatology

Knowledge representation and knowledge engineering are central to classical AI research [25,40]. Machine learning and its sub-field deep learning are foundations of the AI framework. “Machine Learning” refers to the automatic improvement of AI algorithms through experience and massive historical data (training datasets) to build models based on datasets that allow the algorithm to generate prediction and make decisions without programming [41]. “Deep learning” is a division of machine learning founded on artificial neural networks (ANNs) and representation learning. The ANN is a mathematical model that simulates the structure and function of biological neural networks, and an adaptive system with learning capabilities. The performance of an ANN depends on the number and structure of its neural layers and training dataset [42,43]. Deep learning is already widely used to detect and classify skin cancers and other skin lesions [44,45,46]. The most prominent deep learning networks can be divided into recursive neural networks (RvNNs), recurrent neural networks (RNNs), Kohonen self-organizing neural networks (KNNs), generative adversarial neural networks (GANs) and convolutional neural networks (CNNs) [47]. CNNs, a subtype of ANNs, are most frequently used for image processing and detection in medicine, particularly in dermatology, pathology and radiology [48]. Currently, the most implemented CNN architectures in the field of dermatology are GoogleNet, Inception-V3, V4, ResNet, Inception-ResNet V2 and Dense Net [47]. As the raw data source for training CNN architectures for applying deep learning, image sets with a large number of high-quality images are decisive for the diagnostic accuracy, sensitivity and specificity of the final trained AI algorithm [49]. An image set can be used to be managed for image data. The object contains a description of the image, the location of the image and the number of images in the set [50]. The most common image sets used to train AI CAD systems in dermatology today are ISIC archives (2016–2021), HAM10000, BCN20000 and PH2 image sets [51,52,53,54,55,56]. The concepts and components related to AI in the dermatology field are displayed systematically in Table 1.

## 3. Method

This work was carried out by one reviewer (ZXL) and checked by a second reviewer (KCK) in the event of uncertainty.

### 3.1. Search Strategy

A literature search was conducted systematically in three English language electronic databases (PubMed, Web of Science and Google scholar) and three Chinese databases (CQVIP, Wanfang Data and CNKI) to find biomedical and clinical studies of AI and dermatology. We used combinations of terms concerning greenspace (e.g., ‘artificial intelligence’, ‘AI’, ‘AI Algorithm, ‘Deep Learning’, ‘Machine Learning’, ‘Transfer Learning’, ‘Computer Aided Diagnosis’, ‘Meta Data’ ‘Generative Adversarial Networks’ and ‘Convolutional Neural Network’) and dermatology (e.g., ‘dermatology’, ‘dermatoses’, ‘skin lesion’, ‘skin disease’, ‘pigmented skin lesion’, ‘ISIC ’, ‘ISIC challenge’, ‘Melanoma’ and ‘skin cancer’) for the search. Our search was limited to studies written in English, German or Chinese. We also manually searched for a number of studies and other relevant review articles that were included in the references.

### 3.2. Studies Selection

The search results were filtered and only studies that investigated the relationship between AI and dermatology or skin-related surgery were included. Reviews, letters to the editor and clinical research studies were also considered.

### 3.3. Data Extraction

**Figure 2:**
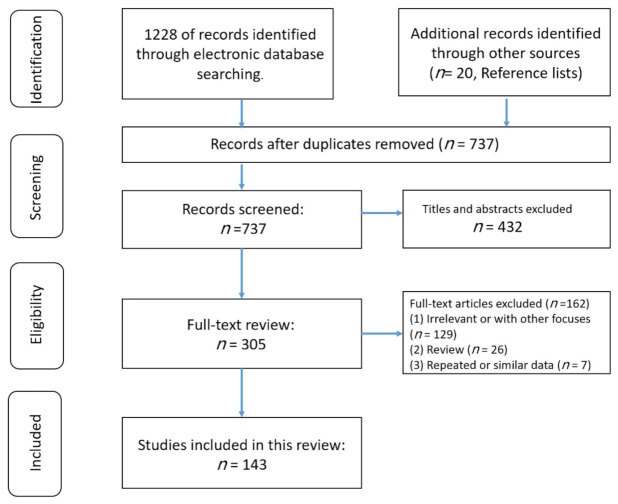
Timeline and major nodes of AI development.

For each study, information on paper (author and publication time), study location, study disease, the type and aim of AI algorithm, image number of learning dataset, outcomes, accuracy, sensitivity and specialty was extracted. A detailed summary of each is provided in Figure 2.

## 4. The Implementation of AI in Dermatology

**Figure 3:**
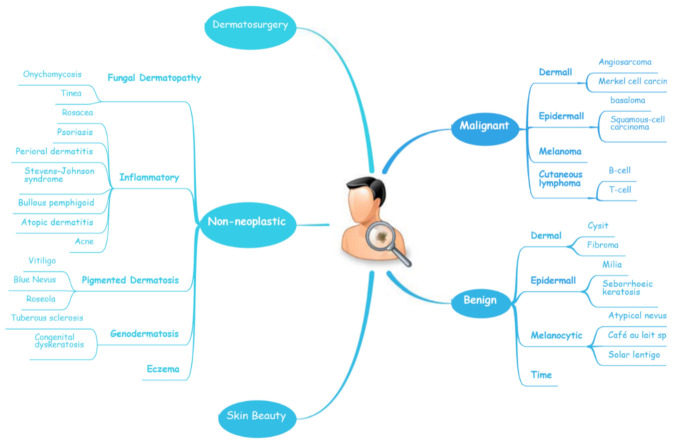
A schematic illustrates the hierarchy of the implementation of AI in dermatology.

**Figure 4:**
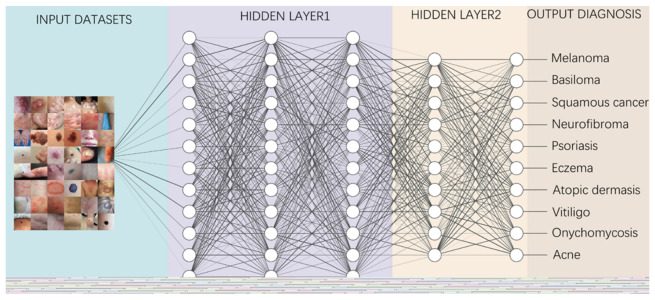
A diagram depicting how to perform classification tasks in an AI neural network.

The diagnosis of skin diseases is mainly based on the characteristics of the lesions [57]. However, there are more than 2000 different types of dermatological diseases, and some skin lesions of different diseases show similarities, which makes antidiastole difficult [58]. At present, the global shortage of dermatologists is increasing with the high incidence of skin diseases. There is a serious deficit of dermatologists and uneven distribution, especially the developing countries and remote areas, which urgently require more medical facility, professional consultation and clinical assistance [59,60]. Rapid iteration in big data, image recognition technology and the widespread use of smartphones worldwide may be creating the largest transformational opportunity for skin diseases’ diagnosis and treatment in this era [61,62]. In addition to addressing the needs of underserved areas and the poor, AI now has the ability to provide rapid diagnoses, leading to more diverse and accessible treatments approaches [63]. An AI-aided system and algorithm will quickly turn out to be normal diagnosis and evaluation-related techniques. The morphological analysis of a lesion is the classic basis of dermatological diagnostics, and the face recognition and aesthetic analysis from AI have also matured and become more reliable [64,65]. Currently, some applications of AI in dermatology have already found their way into clinical practice. Table 2, Table 3 and Table 4 illustrates specific implementation of AI in dermatology visualized with a mind map (Figure 3) [53,66,67]. AI systems based on a deep learning algorithm use plentiful public skin lesion image datasets to distinguish between benign and malignant skin cancers. These datasets contain massive original images in diverse modalities, such as dermoscopy, clinical photographs or histopathological images [68]. In addition, deep learning was used to process the disagreements of human annotations for skin lesion images. An ensemble of Bayesian fully convolutional networks (FCNs) trained with ISIC archive was applied for the lesion image’s segmentation by considering two major factors in the aggregations of multiple truth annotations. The FCNs implemented a robust-to-annotation noise learning scheme to leverage multiple experts’ opinions towards improving the generalization performance using all available annotations efficiently [69]. Currently, the most representative and commonly used AI model is the CNN. It transmits input data through a series of interconnected nodes that resemble biological neurons. Each node is a unit of mathematical operation, a group of interconnected nodes in the network is called a layer and multiple layers build the overall framework of the network (Figure 4) [70,71]. Deep CNNs have also been applied to the automatic understanding of skin lesion images in recent years. Mirikharaji et al., proposed a new framework for training fully convolutional segmentation networks from a large number of cheap unreliable annotations, as well as a small fraction of expert clean annotations to handle both clean and noisy pixel-level annotations accordingly in the loss function. The results show that their spatially adaptive re-weighting method can significantly decrease the requirement for the careful labelling of images without sacrificing segmentation accuracy [72].

Information from the image data set is transmitted through a structure composed of multi-layer connection nodes. Each line is a weight connecting one layer to the next, with each circle representing an input, neuron or output. In convolutional neural networks, these layers contain unique convolutional layers that act as filters. The network made up of many layered filters learn increasingly high-level representations of the image.

### 4.1. AI in Aid-Diagnosis and Multi-Classification for Skin Lesions

#### 4.1.1. Multi-Classification for Skin Lesions in ISIC Challenges

In recent years, the classification of multiple skin lesions has become a hotspot with the increasing popularity of using deep learning algorithms in medical image analysis. Before, metadata indicating information such as site, age, gender, etc., were not included, even though this information is collected by doctors in daily clinical practice and has an impact on their diagnostic decisions. Therefore, the algorithm or AI system that includes this information is better able to reproduce the actual diagnostic scenario, and its diagnostic performance will be more credible. The ISIC challenges consider AI systems that can identify the presence of many different pathologies and provide metadata for labelled cases, thus allowing for a more realistic comparison between AI systems and clinical scenarios. Since the International Skin Imaging Collaboration (ISIC) challenge was held in 2016, it represents the benchmark for diverse research groups working in this area. To date, their database has accumulated over 80,000 labelled training and testing images, which are openly accessible to all researchers and have been used for training algorithms to diagnose and classify various skin lesions [109]. In ISIC 2016–2018, subsets of the image datasets were divided into seven classes: (1) actinic keratosis and intraepithelial carcinoma, (2) basal cell carcinoma, (3) benign keratosis, (4) dermatofibroma, (5) melanocytic nevi, (6) melanoma and (7) vascular skin lesion. From 2019, the atypical nevi were added as the eighth subset. Garcia-Arroyo and Garcia-Zapirain designed a CAD system to participate in ISIC 2016, 2017 Challenge and were ranked 9th and 15th, respectively [110]. In 2018, Rezvantalab et al., investigated the effectiveness and capability of four pre-trained algorithms with HAM10000 (comprising a large part of the ISIC datasets) and PH^2^ state-of-the-art architectures (DenseNet 201, ResNet 152, Inception v3, Inception ResNet v2) in the classification of eight skin diseases. Their overall results show that all deep learning models outperform dermatologists (by at least 11%) [52]. Iqbal et al., proposed a deep convolutional neural network (DNN) model trained using ISIC 2017–2019 datasets that proved to be able to automatically and efficiently classify skin lesions with 0.964 AUR in ROC curve [71]. Similarly, Lucius’ team developed a DNN trained with HM10000 to classify seven types of skin lesions. Statistics showed that the diagnostic accuracy of dermatologists is significantly improved with the help of DNNs [111]. MINAGAWA et al., trained a DNN using ISIC-2017, HAM10000 and Shinshu datasets to narrow the diagnostic accuracy gap for dermatologists facing patients from different regions [112]. Qin et al., established a skin lesion style-based generative adversarial network (GAN) and tested it in the ISIC 2018 dataset, showing that the GAN can efficiently generate high-quality images of skin lesions, resulting in an improved performance of the classification model [73]. Cano et al., applied CNNs based on NASNet architecture trained with a skin image lesion from the ISIC archive for multiple skin lesion classification, which has been cross validated. Its excellent performance suggests that it can be utilized as a novel classification system for multiple classes of skin diseases [74]. Al-masni et al., integrated a deep learning full-resolution convolutional network and a convolutional neural network classifier for segmenting and classifying various skin lesions. The proposed integrated deep learning model was evaluated in ISIC 2016–2018 datasets and achieved an over 80% accuracy in all three for segmentation and discrimination among seven classes of skin lesions, with the highest accuracy of 89.28% in ISIC 2018 [113]. In 2018, Gessert et al., employed an ensemble of CNNs in the ISIC 2018 challenge and achieved second place [53]. Next year, they exploited a set of deep learning models trained with BCN20000 and HAM10000 datasets to solve the skin lesion classification problem, including EfficientNets, SENet and ResNeXt WSL to address the classification of skin lesions and predict unknown classes by analyzing patients’ metadata. Their approach achieved first place in the ISIC 2019 challenge [54].

In recent years, transfer learning technology has also been applied for classifying multiple skin lesions. Transfer learning allows a model developed from one task to be transferred for another task after fine-tuning and augmentation. It is very helpful when we don’t have enough training data sources. When lesion images are difficult to acquire, the algorithmic model can be initially performed with natural images and subsequently fine-tuned with an enhanced lesion dataset to increase the accuracy and specificity of the algorithm, thereby improving the performance on image processing tasks. Singhal et al., utilized transfer learning to train four different state-of-the-art architectures with the ISIC 2018 dataset and demonstrated their practicability for the detection of skin lesions [114]. Barhoumi et al., trained content-based dermatological lesion retrieval (CBDLR) systems using transfer learning, and their results showed that it outperformed a similar CBDLR systems using standard distances [75]. There are also some more studies that have devised AI systems or architectures trained or tested in ISIC datasets and that have gained outstanding performances; we summarize them in detail in Table 2 [23,68,76,77,78,79,80,115,116].

Lately, the ISIC-2021 datasets have just been released. Except for the ISIC 18, ISIC 2019 and ISIC 2020 melanoma datasets, it also contains extra seven datasets with a total of approximately 30,000 images, such as Fitzpatric 17k, PAD-UFS-20, Derma7pt and Dermofit Image. This greatly increases the richness and diversity of the ISIC-2021 archive and correlates the patient’s skin lesion condition with the other disorders of the body, which will provide the basis for the future training of AI algorithms with a more comprehensive and higher diagnostic accuracy. We are also looking forward to the publication of high-quality papers based on this archive [117].

#### 4.1.2. Multi-Classification for Skin Lesions in Specific Dermatosis

In addition to the eight major categories of skin diseases defined in the ISIC challenge, in many specific skin diseases, a differential diagnosis for multiple subtypes is also an urgent issue to be solved. For example, in melanoma, while the common melanoma subtypes superficial spreading melanoma (SSM) and lentigo maligna melanoma (LMM) are relatively easy to diagnose, the morphological features of melanomas on other specific anatomical sites (e.g., mucosa, limb skin and nail units) are often overlooked [81]. On top on that, some benign nevus of melanocytic origin can also be easily confused with malignant melanoma in morphology [118]. Among the common pigmentation disorders, many are caused by abnormalities in melanin in the skin. Although they are similar in appearance, they are diseases with different pathological structures and treatment strategies. Diagnostic models based on AI algorithms can improve the diagnostic accuracy and specificity of these diseases so as to benefit dermatologists by reducing the time and financial cost of the diagnosis [119].

##### Melanocytic Skin Lesions

Since Binder’s team applied an ANN to discriminate between benign naevi and malignant melanoma in 1994, increasing numbers of AI algorithms are employed for the multi-classification of melanocytic skin lesions [82]. Moleanalyzer pro is a proven commercial CNN system for the classification of melanogenic lesions. Winkler and his team used the system, which was trained with more than 150,000 images, to investigate its diagnostic performance across different melanoma localizations and subtypes in six benign/malignant dermoscopic image sets. The CNN showed a high-level performance in most sets, except for the melanoma in mucosal and subungual sites, suggesting that the CNN may partly offset the impact of a reduced human accuracy [81]. In two studies by HA Haenssle et al., in 2018 and 2020, CNNs were also used in comparison with specialist dermatologists to detect melanocytic/non-melanocytic skin cancers and benign lesions. In 2018, the CNN trained with Google’s Inception v4 CNN architecture was compared with 58 physicians. The results showed that most dermatologists outperformed the CNN, but the CNN ROC curves revealed a higher specificity and doctors may benefit from assistance by a CNN’s image classification [55]. In 2020, Moleanalyzer pro was compared with 96 dermatologists. Even though dermatologists accomplish better results when they have richer clinical and textual case information, the overall results show that the CNN and most dermatologists perform at the same level in less artificial conditions and a wider range of diagnoses [56]. Sies et al., utilize the Moleanalyzer pro and Moleanalyzer daynamole systems for the classification of melanoma, melanocytic nervus and other dermatomas. The results showed that the two market-approved CAD systems offer a significantly superior diagnostic performance compared to conventional image analyzers without AI algorithms (CIA) [83].

##### Benign Pigmented Skin Lesions

Based on a wealth of experience and successful clinical practice, scholars have gradually tried to apply AI to differentiate a variety of pigmented skin diseases with promising results. Lin’s team pioneered the use of deep learning to diagnose common benign pigmented disorders. They developed two CNN models (DenseNet-96 and ResNet-152) to identify six facial pigmented dermatoses (the nevus of Ota, acquired nevus of Ota, chloasma, freckles, seborrheic keratosis and cafe-au-lait spots).Then, they introduced ResNet.99 to build a fusion network, and evaluated the performance of the two CNN with fusion networks separately. The results showed that the fusion network performance was the best and could reach a level comparable to that of dermatologists [84]. In 2019, Tschandl et al., conducted the world largest comparison study between the machine-learning algorithm and 511 dermatologists for the diagnosis accuracy of pigmented skin lesion classification. The algorithm was, on average, 2.01% more correct in its diagnosis compared to all human readers. The result disclosed that machine-learning classifiers outperform dermatologist in the diagnosis of skin pigmented lesions and should be more widely used in clinical practice [120]. In the latest study, Lyakhov et al., established a multimodal neural network for the hair removal preliminary process and differentiation of the 10 most common pigmented lesions (7 benign and 3 malignant). They found that fusing metadata from various sources could provide additional information, thereby improving the efficiency of the neural network analysis and classification system, as well as the accuracy of the diagnosis. Experimental results showed that the fusion of metadata led to an increase in recognition accuracy of 4.93–6.28%, with a maximum diagnosis rate of 83.56%. The study demonstrated that the fusion of patient statistics and visual data makes it possible to find extra connections between dermatoscopic images and medical diagnoses, significantly improving the accuracy of neural network classification [85].

##### Inflammatory Dermatoses

Inflammatory dermatoses are a group of diseases caused by the destruction of skin tissue as a result of immune system disorders, including eczema, atopic dermatitis, psoriasis, chronic urticarial and pemphigus. Newly recorded histological findings and neoteric applications of immunohistochemistry have also refined the diagnosis of inflammatory skin diseases [121]. AI CAD systems are able to optimize the workflow of highly routinely diagnosed inflammatory dermatoses. A multi-model, multi-level system using an ANN architecture was designed for eczema detection. This system is conceived as an architecture with different models matching input features, and the output of these models are integrated through a multi-level decision layer to calculate the probability of eczema, resulting in a system with a higher confidence level than a single-level system [86]. From 2017 onwards, neural networks have been shown to be useful for diagnosing acne vulgaris [90]. The latest publications on the use of computer-aided systems in acne vulgaris are based on a wealth of data from cell phone photographs of affected patients, which enable the development of AI-based algorithms to determine the severity of facial acne and to identify different types of acne lesions or post-inflammatory hyperpigmentation [91]. Scientists in South Korea trained various image analysis algorithms to recognize images of fungal nails. For this purpose, they used datasets of almost 50,000 nail images and 4 validation datasets of a total of 1358 images. A comparison of the respective diagnostic accuracy (measured in this study by the Youden index) of differently trained assessors and the AI algorithm showed the highest diagnostic accuracy in the computer-based image analysis and was significant superior to dermatologists (*p* = 0.01) [87].

### 4.2. AI in Aid-Diagnosis and Binary-Classification for Specific Dermatosis

#### 4.2.1. Skin Cancer

The incidence of skin cancer has been increasing yearly [58,122]. Although its mortality rate is relatively low [123], it remains a heavy economic burden on health services and can cause severe mental problems, especially as most skin cancers occur in highly visible areas of the body [124]. Due to the low screening awareness, a lack of specific lesion features in early skin cancer and insufficient adequate clinical expertise and services, most patients were only diagnosed at an advanced stage, thus leading to a poor prognosis [124,125], so there is an urgent need for AI systems to help clinicians in this field.

##### Melanoma

Melanoma is the deadliest type of skin cancer. The early screening and early diagnosis of melanoma is essential to improve patient survival [126]. Currently, dermatologists diagnose melanoma mainly by applying the ABCD principle based on the morphological characteristics of melanoma lesions [127]. However, even for experienced dermatologists, this manual examination is non-trivial, time consuming and can be easily confused with other benign skin lesions [128]. Thus, most AI-driven skin cancer research has focused on the classification of melanocytic lesions to aid melanoma screening. In 2004, Blum et al., pioneered the use of computer algorithms for the diagnosis of cutaneous melanoma and proved that a diagnostic algorithm for the digital image analysis of melanocytic diseases could achieve a similar accuracy to expert dermatoscopy [88]. In 2017, Esteva et al., trained a GoogleNet-Inception-v3-based CNN with the training dataset, including 129,450 clinical images of 2032 different diseases from 18 sites. The performance of the CNN was compared with 21 dermatologists in two critical binary classifications (the most common cancer and the deadliest skin cancer) of biopsy-confirmed clinical images. The CNN’s performance on both tasks was competent, and comparable to that of dermatologists, demonstrating its ability to classify skin cancer [66]. The ISIC Melanoma Project has also created a publicly accessible archive of images of skin lesions for education and research. Marchetti et al., summarized the results of a melanoma classification for ISIC challenge in 2016, which involved 25 competing teams. They compared the algorithm’s diagnosis with those of eight experienced dermatologists. The outcomes showed that automated algorithms significantly outperformed the dermatologists in diagnosing melanoma [89]. Subsequently, they made a comparison of the computer algorithms’ performance of 32 teams in the ISIC 2017 challenge with 17 human readers. The results also demonstrated that deep neural networks could classify skin images of melanoma and its benign simulants with a high precision and have the potential to boost the performance of human readers [22]. Filho and Tangs’ team have utilized the ISIC 2016, 2017 challenge and PH2 datasets to develop the algorithm for the classification and segmentation of the melanoma area automatically. Their test outcomes indicated that these algorithms could dramatically improve the doctors’ efficiency in diagnosing melanoma [51,129]. In MacLellan’s study, three AI-aid diagnosis systems were compared with dermatologists using 209 lesions in 184 patients. The statistics showed that the Moleanalyzer pro had a relative high sensitivity and the highest specificity (88.1%, 78.8%), whereas local dermatologists had the highest sensitivity but a low specificity (96.6%, 32.2%) [130]. Consistently, Moleanalyzer pro also showed its reliability in the differentiation of combined naevi and melanomas [131]. It is also possible for dermatologists to build a whole-body map using a 3D imaging AI system; its application is of particular relevance in the context of skin cancer diagnostics. The 360° scanner uses whole-body images to create a “map” of pigmented skin lesions. Using a dermatoscope, atypical and altered nevi can also be examined microscopically and stored digitally. With the help of intelligent software, emerging lesions or lesions that change over time are automatically marked during follow-up checks—an important feature for recognizing a malignancy and initiating therapeutic measures [132]. In addition, in the long term, high-risk melanoma populations will benefit from a clinical management approach that combines an AI-based 3D total-body photography monitor with sequential digital dermoscopy imaging and teledermatologist evaluation [133,134].

##### Non-Melanoma Skin Cancer

AI is also widely used to differentiate between malignant and benign skin lesions, along with the detection of non-melanoma skin cancer (NMSC). Rofman et al., proposed a multi-parameter ANN system based on personal health management data that can be used to forecast and analyze the risk of NMSC. The system was trained and validated by 2056 NMSC and 460,574 non-cancer cases from the 1997–2015 NHIS adult survey data, and was then further tested by 28058 individuals from the 2016 NHIS survey data. The ANN system is available for the risk assessment of non-melanoma skin cancer with a high sensitivity (88.5%). It can classify patients into high, medium and low cancer risk categories to provide clinical decision support and personalized cancer risk management. The study’s model is therefore a prediction, where clinicians can obtain information and the patient risk status to detect and prevent non-melanoma skin cancer at an early stage [94]. Alzubaidi et al., propose a novel approach to overcome the lack of enough input-labeled raw skin lesion images by retraining a deep learning model based on large unlabeled medical images on a small number of labeled medical images through transfer learning. The model has an F1-score value of 98.53% in distinguishing skin cancer from normal skin [95].

##### Neurofibroma

Neurofibromatosis (NF) is a group of three conditions in which tumors grow in the nervous system, and are NF1, NF2 and schwannomatosis [135]. NF1 is the most common neurofibroma and cancer susceptibility disease. Most patients with NF1 have a normal life expectancy, but 10% of them develop malignant peripheral nerve sheath tumors (MPNST), which is a major cause of morbidity [136]. Therefore, the timely differentiation of benign and malignant lesions has direct significance for improving the survival rate of patients. Wei et al., successfully established a Keras-based machine-learning model that can discriminate between NF1-related benign and malignant craniofacial lesions with a very high accuracy (96.99 and 100%) in validation cohorts 1 and 2 and a 51.27% accuracy in various other body regions [137]. Plexiform neurofibroma (PN) is a prototypical and most common NF1 tumor. Ho et al., created a DNN algorithm to conduct a semi-automated volume segmentation of PNs based on multiple b-value diffusion-weighted MRI. They evaluated the accuracy of semi-automated tumor volume maps constructed by a DNN compared to manual segmentation and revealed that the volumes generated by the DNN from multiple diffusion data on PNs have a good correlation with manual volumes, and that there is a significance between PN and normal tissue [97]. Interestingly, Bashat and his colleagues also demonstrated that a quantitative image representation method based on machine learning may assist in the classification between benign PNs and MPNST in NF1 [102]. In a similar initiative, Duarte et al., used grey matter density maps obtained from magnetic resonance (MR) brain structure scans to create a multivariate pattern analysis algorithm to differentiate between NF1 patients and healthy controls. A total of 83% of participants were correctly classified, with 82% sensitivity and 84% specificity, demonstrating that multivariate techniques are a useful and powerful tool [103].

#### 4.2.2. Application of AI for Inflammatory Dermatosis

##### Psoriasis

The prevalence of psoriasis is 0% to 2.1% in children and 0.91% to 8.5% in adults [138]. The psoriasis area and severity index (PASI), body surface area (BSA) and physician global assessment (PGA) are the three most commonly used indicators to evaluate psoriasis severity [139,140]. However, both PASI and BSA have been repeatedly questioned for their objectivity and reliability [141]. It would therefore be of great help to use AI algorithms to make a standardized and objective assessment. Nowadays, machine-learning-based algorithms are available to determine BSA scores. Although this algorithm had slight limitations in detecting flaking as diseased skin, it has reached an expert level in BSA assessment [104]. At present, there are already computer-assisted programs for PASI evaluation, which, however, still require human assistance and function by recognizing predefined threshold values for certain characteristics [98]. Another study by Fink’s team is also based on image analysis with the FotoFinder^TM^. The accuracy and reproducibility of PASI has been impressively improved with the help of semi-automatic computer-aided algorithms [99]. These technological advances in BSA and PASI measurements are expected to greatly reduce the workload of doctors while ensuring a high degree of repeatability and standardization. In addition to the three above indicators, Anabik Pal et al., used erythema, scaling and induration to build a DNN to determine the severity of psoriatic plagues. The algorithm is given a psoriasis image and then makes a prediction about the severity of the three parameters. This task is seen as a new multi-task learning (MTL) problem formed by three interdependent subtasks in addition to three different single task learning (STL) problems, so the DNN is trained accordingly. The training dataset consists of 707 photographs and the training results show that the deep CNN-based MTL approach performs well when grading the disease parameters alone, but slightly less well when all three parameters are correctly graded at the same time [142].

AI can also assist in evaluating and diagnosing psoriasis. Munro’s microabscesses (MM) is a sign of psoriasis. Anabik Pak et al., presented a computational framework (MICaps) to detect neutrophils in the epidermal stratum corneum of the skin from biopsy images (a component of MM detection in skin biopsies). Using MICaps, the diagnosis performance was increased by 3.27% and model parameters were reduced by 50% [143]. A CNN algorithm that differentiated among nine diagnoses based on photos made fewer misdiagnoses and had a lower omission diagnostic rate of psoriasis compared to 25 dermatologists [92]. In addition, Emma et al., used machine learning to find out which psoriasis patient characteristics are associated with long-term responses to biologics [144]. Thanks to AI, an amelioration in diagnosis and treatment can be inferred in psoriasis patients.

##### Eczema

The challenge in the computer-aided image diagnosis of eczematous diseases is to correctly differentiate not only between disease and health, but also between different forms of eczema. The eczema stage and affected area are the most essential factors in effectively assessing the dynamics of the disease. It is not trivial to accurately identify the eczema area and other inflammatory dermatoses on the basis of photographic documentation. The macroscopic forms of eczema are diverse, with different stages and varying degrees of distribution and severity [145]. The prerequisite for training algorithms for the AI-supported image analysis of all of these various assessment parameters is therefore a correspondingly large initial quantity of image files that have been optimized and adjusted in terms of the recording technology. Forms of eczema with disseminated eruption, such as the corresponding manifestation patterns of atopic dermatitis, would also be linked to the availability of automated digital, serial whole-body photography for an efficient and time-saving AI-supported calculation of an area score. Han et al., trained a deep neural-network-based algorithm. The algorithm is able to differentiate between eczema and other infectious skin diseases and to classify very rare skin lesions, which has direct clinical significance, and to serve as augmented intelligence to empower medical professionals in diagnostic dermatology. They even showed that treatment recommendations (e.g., topical steroids versus antiseptics) could also be learned by differentiating between inflammatory and infectious causes. It remains to be seen and questioned, however, whether an AI-aided severity assessment and a clinically practicable area score can be derived from this as a prerequisite for a valid follow-up in the case of eczema [93]. Schnuerle et al., designed a support-vector-machine-based image processing method for hand eczema segmentation with industry swiss4ward for operational use at the University Hospital Zurich. This system uses the F1-score as the primary measurement and is superior to a few advanced methods that were tested on their gold standard dataset likewise [100]. Presumably, a combination of such an AI-aided image analysis and molecular diagnostics can optimize the future differential diagnostic classification of eczema diseases, as recently predicted for various clinical manifestations of hand dermatitis [146].

##### Atopic Dermatitis

Atopic dermatitis (AD) is the most common chronic inflammatory disease, with a prevalence of 10% to 20% in developed countries [147]. It usually starts in childhood and recurs multiple times in adulthood, greatly affecting patients’ quality of life [148]. In 2017, Gustofson’s team identified patients with AD via a machine-learning-based phenotype algorithm. The algorithm combined code information with the collection of electronic health records to achieve a high positive predictive value and sensitivity. These results demonstrate the utility of natural language processing and machine learning in EHR-based phenotyping [105]. An ANN algorithm was developed to assess the influence of air contaminants and weather variation on AD patients; their results proved that the severity of AD symptoms was positively correlated with outdoor temperatures, RH, precipitation, NO_2_, O_3_ and PM10 [149]. In the latest study, a fully automatic approach based on CNN was proposed to analysis multiphoton tomography (MPT) data. The proposed algorithm correctly diagnosed AD in 97.0 ± 0.2% of all images presenting living cells, with a sensitivity of 0.966 ± 0.003 and specificity of 0.977 ± 0.003, indicating that MPT imaging can be combined with AI to successfully diagnose AD [96].

##### Acne

The assessment of AI has been very effective. Melina et al., showed an excellent correlation between the automatic and manual evaluation of the investigator’s global assessment with r = 0.958 [150]. In the case of acne vulgaris in particular, such a procedure could prevent far-reaching consequences with permanent skin damage in the form of scars.

##### Vitiligo

The depigmented macules of vitiligo are usually in high contrast to unaffected skin. Vitiligo is more easily recognized by AI systems than features of eczema or psoriasis lesions with poorly defined borders. Computer-based algorithms used for the detection of vitiligo with an F1 score of 0.8462 demonstrated an impressive superiority to pustular psoriasis [151]. Luo designed a vitiligo AI diagnosis system employing cycle-consistent adversarial networks (cycle GANs) to generate images in Wood’s lamp and improved the image resolution via an attention-aware dense net with residual deconvolution (ADRD). The system achieved a 9.32% improvement in classification performance accuracy compared to direct classification of the original images using Resnet50 [106]. Makena’s team built a CNN that performs vitiligo skin lesion segmentation quickly and robustly. The network was trained on 308 images with various lesion sizes, intricacies and anatomical locations. The modified network outperformed the state-of-the-art U-Net with a much higher Jaccard index score (73.6% versus 36.7%) and shorter segmentation time than the previously proposed semi-autonomous watershed approach [107]. These novel systems have proved promising for clinical applications by greatly saving the testing time and improving the diagnostic accuracy.

##### Fungal Dermatosis

Gao et al., invented an automated microscope for fungal detection in dermatology based on deep learning. The system is as proficient as a dermatologist in detecting skin and nail specimens, with sensitivities of 99.5% and 95.2% and specificities of 91.4% and 100%, respectively [101].

### 4.3. Application of AI for Aesthetic Dermatology

AI combined with new optical technologies is also increasingly being applied in aesthetics dermatology. Examples include face recognition, automatic beautification in smartphones and related software. So-called smart mirror analyzers are now available on the Internet, which are AI-assisted technologies with image recognition systems that analyze the skin based on its appearance and current external environment and recommend skin care products accordingly [152]. The program ArcSoft Protrait can automatically identify the wrinkles, moles, acne and cicatrice and intelligently soften, moisturize and smooth the skin while retaining a maximum skin texture and detail, greatly simplifying the cumbersome and time-consuming portrait process [61,64,153]. AI also plays an essential role in facial aesthetics assessing. For this purpose, ANNs are trained using face image material that people judge independently according to aesthetic criteria based on various criteria. The ANN learns from photos and their respective attractiveness ratings to make human-like judgments about the aesthetics of the face [65]. New applications objectively evaluate each photo on the basis of over 80 facial coordinates and nearly 7000 associated distances and angles [108] (Table 4).

### 4.4. Applications of AI for Skin Surgery

Radical resection and amputation are the best means of preventing recurrence and fatal metastasis for malignant dermatoma [154]. A skin or flap graft via microsurgery and the application of prosthesis play a crucial role in improving the quality of life of patients after resection [155,156]. Adequate microvascular anastomosis is the key to a successful microvascular-free tissue transfer. As a basic requirement in this regard, the surgeon must have excellent microsurgery skills. Thanks to the support of a series of auxiliary equipment such as microscopes, magnifications of up to 10 to 15 times are possible and allow for the anastomosis of small vessels. Nevertheless, due to physiological tremor, only vessels of up to approximately 0.5–1 mm in size can be safely anastomosed, especially in lymphatic surgery or perforator-based flaps, where the vascular caliber may even be smaller, which is why surgeons reach their limits here [157]. In this background, the expansion of surgical microscopes to include robotics and AI capabilities represents a promising and innovative approach for surpassing the capabilities of the human hand. The aim is to use robots equipped with AI to eliminate human tremor and to enable motion scaling for an increased precision and dexterity in the smallest of spaces [158]. By downscaling human movements, finer vessels can be attached. In the future, advances could be achieved in the field of ultra-microsurgery and anastomoses in the range of 0.1–0.8 mm on the smallest vessels or nerve fascicles. In the long term, intelligent robotics could also automate technically demanding tasks, such as microsurgical anastomosis performed by robots, or the implementation of a real-time feedback system for the surgeon.

Prosthetics have also evolved with the implementation of AI. After amputation injuries, prostheses can now restore not only the shape but also essential functions of the amputated extremity; in this way, they make a significant contribution to the reintegration of the patient into society. The mental control of the extremity remains in the brain even after amputation. When movement patterns are imagined, despite the lack of end organs to perform them, neurons will still transmit corresponding nerve signals [159]. Prostheses can now receive the electrical potential via up to eight electrodes and assign them to the respective functions via pattern recognition and innovative technological methods equipped with AI, and can ensure that patients better use the prosthesis in their daily lives [160,161]. This enables the patient to directly control different grip shapes and movements, which means that gripping movements can be realized much faster and more naturally in terms of movement behavior.

The application of AI-based surgical robots in skin surgery is now also becoming widespread. Compared to traditional open surgery, robotic-assisted surgery offers 3D vision systems and flexible operating instruments, with potentially fewer postoperative complications as a result. In 2010, Sohn et al., first applied this technique to treat two pelvic metastatic melanoma patients [162]. In 2017, Kim successfully treated one case of vaginal malignant melanoma using robotic-assisted anterior pelvic organ resection with ileoccystostomy [15]. One year later, Hyde successfully treated four cases of malignant melanoma using robotic-assisted inguinal lymph node dissection [163]. Miura et al., found that robotic assistance provided a safe, effective and minimally invasive method of removing a pelvic lymph from patients with peritoneal metastases melanoma, with shorter hospital stays compared to normal open surgery [164]. Medical robots are also involved in the field of hair transplantation. In 2011, the ARTAS system was officially approved by the US-FDA for male hair transplantation, providing clear and detailed characteristics of the donor area by capturing microscopically magnified images and computer-aided parameters to facilitate the acquisition of complete follicular units from the donor area [16]. The system reduces labor consumption and eliminates human fatigue and potential errors, and the procedure time is significantly reduced [165].

## 5. Computer-Aided Dermatology AI Systems on Market

With the rapid development of AI over the past decade, a number of ‘skin’ medical systems and instruments with multiple applications have been commercialized. These systems and instruments have ample image datasets to assist in skin examination, monitoring the skin condition, clinical follow-up and providing treatment advice or guidance. Here, we briefly summarize the most widely used dermatology AI systems and smartphone apps of the last 15 years (Table 5).

As a state-of-the-art full-body scanning imaging and intelligent identification system, the Vectra WBS360 allows the entire skin surface to be acquired with a macroscopic quality resolution through a single capture. Clinicians can map and survey pigmented lesions and distributed dermatoses with integrated software. Other applications include documenting pigmented lesions, psoriasis and vitiligo with the help of 3D imaging systems that allow for detailed documentation and organization of pre- and post-operative image records. Its companion dermoscope VEOS DS3 combines optics and illumination with wireless capture. The AI-based DermaGraphix imaging software also helps in assessing the risk of the lesion’s malignancy: it allows physicians to label and monitor lesions and process images in a protected and implementable image management system [132,133,134].

Another AI skin system from Canfield, VISIA has been in the market for over 15 years and has evolved into its seventh generation. The system uses cross-polarization and UV illumination to record and measure surface and sub-surface skin conditions. Canfield’s RBX^®^ technology isolates the distinctive color characteristics that lead to the red and brown skin components of color concentration, such as spider veins and hyperpigmentation. Its new AI wrinkle algorithm dramatically increases the detection and precision of fine lines and wrinkles. It can also simulate the effect of each region after injecting different volumes, and can simulate how patients might appear from the ages of 18–80. It provides a finer visualization of sub-surface melanin and vascularity conditions for all skin types and ethnicities. In addition, it allows for the grading of patients’ skin using the world’s largest database of skin characteristics, and measures blemishes, wrinkles, texture, pores, UV spots, red areas and porphyrins [166,167]. A study assessing the clinical value of VISIA suggests that 86% of respondents agreed that VISIA analysis had improved their understanding of and attention toward their skin health. They would all recommend VISIA analysis to other people and 62% of them preferred a clinical practice with a VISIA system [168].

An AI system specifically designed to identify skin cancer, FotoFinder, debuted in 1991. It performs skin cancer diagnosis through automated whole-body mapping and digital dermoscopy, as well as psoriasis documentation and aesthetic imaging. In addition, FotoFinder systems are used in daily practice and related studies. Its AI-based software Moleanalyzer pro, working with deep learning algorithms, allows for a risk-of-malignancy evaluation. It is a market-approved CNN and currently has the largest dataset of dermoscopic images, including their associated diagnosis. The CNN has already been involved in several comparative studies in skin lesions diagnosis, and its reliability and feasibility have been recognized [55,56,81,83,130,131]. Dermascan is also a medical imaging system focusing on monitoring and differential diagnostics in skin cancer. It uses polarization to capture the skin surface and automatically analyzes traces of hyperpigmentation. All patient and localization-related images are saved in a database and linked to the video–dermoscopy system. By using digital photo documentation, the system can identify emerging pigmentation marks and diagnose changes in existing lesions [169].

Miravex’s Antera 3D imaging system is a device and software complex with powerful and versatile data handling and consultation tools for the analysis and qualitative measurement of wrinkles, texture, pigmentation, redness and other various dermatologic conditions. Antera 3D uses an AI algorithm to reconstruct full 3D images of the skin surface and is particularly suitable for the analysis of topographical features such as wrinkles, skin texture, pores and volume. For morphological analysis (wrinkles, texture, volumes, etc.), tests on artificial skin samples under controlled conditions have established an instrumental error of less than 2%, demonstrating the high level of measurement reproducibility offered by the Antera 3D camera [170,171,172].

Following the commercially available artificial intelligence skin system, AIDERMA was born as the first AI-assisted comprehensive platform for the diagnosis and treatment of skin diseases. With leading AI image recognition technology as its core, AIDERMA provides doctors with integrated support for assisting diagnosis, case management, professional education and patient management, helping doctors to improve their diagnosis and treatment efficiency in all aspects and escorting them in their clinical work. AIDERMA can intelligently identify skin lesion photos and give the names of skin diseases directly. In the competition with the FotoFinder system in 2018, its diagnosis accuracy rate reached 80%. Smart skin is now open to Chinese certified physicians and can identify 90 types of diseases with an average accuracy rate of 86%. The product has been clinically tested in more than 3400 hospitals since its launch, helping doctors complete nearly 80,000 auxiliary diagnoses and supporting them to access over three million clinical contents [173,174].

Out of complex and large AI systems and platforms, some light-weight AI-based dermatology diagnostic apps for smart phones have also recently emerged. Dermacompass by swiss4ward is a learning tool for dermatologists. It contains skin disease images along with treatment algorithms and also provides an individual case diagnosis and image comparison. This app uses automatic image analysis to grade the medical severity of hand eczema and detects hand eczema through computer vision and machine learning. DermoScanner is an application leveraging the power of AI and deep learning and allows users to analyze skin moles and detect skin cancers via a mobile camera [61,153,175].

**Table 5:** On market aid-dermatology AI system and apps.

Name	Manufacturer	Country	On Market Year	Platform	Application	Reference
Moleanalyzer pro	Fotofinder	Germany	2018	Windows	Analyzes melanocytic as well as non-melanocytic skin lesions and calculates an AI score for mole risk assessment	[97,137]
Vectra WBS 360	Canfiield	USA	2017	Windows	Capturing the entire skin surface in macro quality resolution with a single capture, to identify and monitors pigment lesions automatically or mannually	[102,103,138]
Visia skin	Canfiield	USA	2007	Windows	Capturing key visual information for eight areas affecting complexion health and appearance and to provide an informative comparison of patient’s complexion’s characteristics to others of same age and skin type	[173,174,175]
Antera 3D	Miravex	Ireland	2011	Windows	Analysis and measurement of wrinkles, texture, pigmentation, redness and other lesions	[176]
Dermoscan X2	Dermoscan	Germany	2017	Windows	Identification of the new or modified lesions with digital photo documentations and makes automatic comparison of pigmentation marks	[177]
AIDERMA	Dingxiangyuan	China	2018	Online	Automatic identification of skin disorders and stores patient’s medical record in the cloud safely	[178,179]
DermEngine	MetaOptima Technology Inc.	Canada	2015	Android and iOS	Imaging, documentation and analysis of skin conditions including skin cancer; offers business intelligence features designed for practice management	[71]
Skin-App	Swiss4ward	Switzerland	2017	Android and iOS	Detection of hand eczema automatically	[71]
Neurodermitis Helferin|Nia	Nia Health	Germany	2019	Android and iOS	Marks affected areas on the clear body diagram, takes photos and documents of the current severity of the neurodermatitis and gives personalized suggestions after further analysis	[157]
DermoScanner	Neat Technology lnc.	N/A	2019	Android	Analysis of skin moles and detects skin cancers at an early stage when it is most treatable.	[159]
Dermacompass	Swiss4ward	Switzerland	2017	Android and iOS	It contains skin diseases, pictures and algorithms for treatment and provides individual case diagnosis and image comparison for dermatologists	[180]

## 6. Attitudes of Different Groups of People towards AI in Dermatology

**Figure 5:**
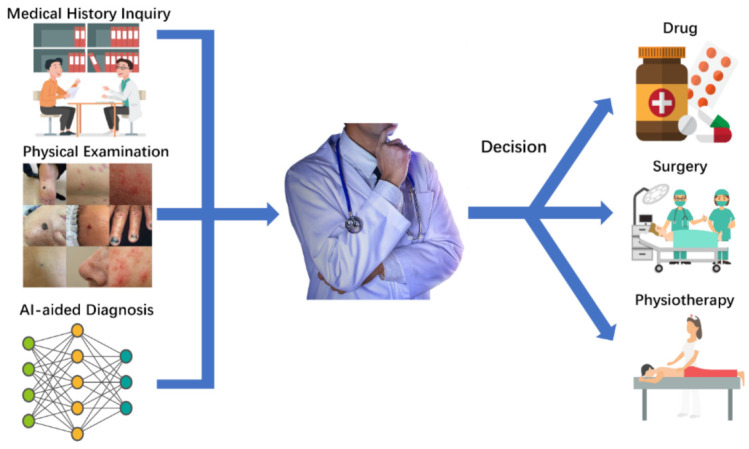
The schematic diagram shows the hypothetical use of machine learning algorithms to help dermatologists diagnose lesions to make appropriate clinical decisions. An emerging AI model CNN can help non-expert clinicians narrow the range of differential diagnosis and provide appropriate treatments.

In recent years, the application of AI in medical image recognition and dermatology has become increasingly intensive and broader. AI has also gradually become a hot topic of discussion in dermatology and dermatopathology. The current health care society and legal framework are more suitable for using AI as a decision aid for dermatologists, especially in terms of assisting the diagnosis (Figure 5). On account of the rapid development of AI and its already widespread use by patients and doctors, several international and regional survey studies were conducted. From January to June 2019, 1271 people from 92 countries were surveyed via an online questionnaire. Respondents identified dermoscopic images as the mightiest potential application of AI in dermatology. A total of 77.3% approved or strongly approved that AI would strengthen dermatology and 79.8% used AI as a part of medical training. In comparison, only 5.5% (70 of 1271) agreed or strongly agreed that dermatologists will be replaced by AI in the foreseeable future [176]. Following an international survey of dermatopathologists from the same research team, responses were received from 718 people, which included 91 countries. In general, 72.3% of respondents agreed or strongly agreed that AI will improve dermatopathology and 84.1% thought that AI should be part of medical training. Only 6.0% of the responders agreed that the human pathologist will be replaced by AI in the future. Concerning diagnosis classification, the automated detection of mitosis had the highest potential (79.2%) and 42.6% felt that automated recommendations for skin tumor diagnosis had strong or very strong potential [181]. Compared to doctors, most patients know less about AI. A qualitative study using semi-structured interview analysis and recruiting 48 patients was conducted from May to July 2019. A total of 60% participants cited an improved diagnostic speed and access to healthcare as the most common advantages of AI for skin cancer monitoring. An increased patient anxiety was the most common risk (40%). Patients identified more precise diagnoses (33 [69%]) and less precise diagnoses (41 [85%]) as the greatest advantages and disadvantages of AI, respectively. A total of 36 patients (75%) would recommend AI to family and friends [182].

In summary, both dermatologists and pathologists are generally optimistic about the impact and potential benefit of AI in dermatology. However, only a minority had either good or excellent knowledge of AI. Most dermatologists believe that it will improve our diagnostic capabilities and most pathologists deemed that the greatest potential of AI is expected for narrowly specified tasks rather than global automated diagnostic recommendations. A minority of dermatologists and pathologists are concerned about being replaced by AI in the foreseeable future. Patients appear willing to use AI for skin cancer monitoring if applied in a way that maintains the integrity of the human doctor–patient relationship.

## 7. Current Limitations of the Application of AI in the Field of Dermatology

At present, there are several major challenges toward the application of AI in the medical field: (1) a small sample size and inadequate quality manual annotation. The current training datasets for AI algorithms is insufficient and there is also a lack of plentiful experienced doctors involved in the identification and labelling of samples. This deficiency directly leads to the accuracy and practicability of AI algorithms not meeting the needs of daily clinical applications [177]; (2) the disjunction between AI algorithms and actual medical requirements and application scenarios. Due to the lack of sufficient high-quality training sets, AI algorithms and applications are generally only developed based on existing samples. Unlike human doctors, AI cannot be upgraded and updated with the vast experience gained over time. As a result, they are unable to meet the growing clinical and scientific demands of reality. Certain specific locations, such as hairy scalp and mucous membranes and rare skin conditions, currently remain a limitation for AI recognition. The accuracy of ANNs is currently also restricted by image artifacts, such as colored markings on the skin, including tattoos [178]. (3) The variety of diseases in dermatology and the lack of uniform criteria for identification and diagnosis make it difficult to teach AI how to identify and diagnose multiple skin diseases [179]. Currently, AI is more commonly used to distinguish between normal and abnormal. A bottleneck still exists for the use of AI for the automatic recognition and diagnosis of multiform dermatopathological images [180,183]. In addition, there are rare diseases in dermatology, where the number of cases is very small and the amount of specimens is not sufficient to provide the necessary training for the machine learning, which is also a major challenge for AI in dermatology [184].

## 8. Future Trends of Artificial Intelligence in Medical Field and Dermatology

Al models, computing power and big data are the three cornerstones for the development of AI technology [185]. The deep learning algorithm, represented by the ANN, has become the core engine of AI application. In the dermatological sector, as we have summarized above, AI can provide better patient care as well as diagnosis and medical imaging interpretation; its technology can screen for various diseases more accurately and effectively [21,22,29,30,31,32,33,34,35,36,37,38,39]. The application of AI and related technologies in public health is evolving rapidly since the first computer-aided systems were still built up by humans in the 1990s [82]. In recent years, the use of AI-aided systems and deep learning processes with ANNs has been further developed. In the era of big data, swarm intelligence, cross-media intelligence, human–machine hybrids and enhanced augmented autonomous intelligence systems are five new trends for the prospective evolution of AI [186,187].

As technology advances, AI is constantly expanding its subdivisions that can be applied, and shows five trends for future development in the medical and dermatological field.

**The first trend is** the increasing support of AI diagnostic platforms for dermatologists. AI imaging systems can reduce the physician workload, and many AI systems have achieved an accuracy comparable to doctors in the diagnosis of pigments lesions. Many of the studies above show that AI has an accuracy in well-defined tasks comparable to that of human doctors, and a greater efficiency. In a wide range of tasks with dichotomous inputs and outputs, AI typically has a higher sensitivity and specificity than dermatologists, and can also identify more subtle lesion locations [66,188,189]. In the face of multi-classification diagnoses that are more closely aligned with clinical scenarios, AI algorithms have also proven their dermatologist-level accuracy [129,190].

**The second trend** is the emergence of a new generation of intelligent medical devices and instruments. The development of AI is not limited to stand-alone software, but a large amount of hardware has also undergone disruptive changes. In the comparative studies mentioned above, systems such as FotoFinder, Canfield Vectra WBS360 and Antera 3D have been applied in several hospitals and have proven their reliability. These devices offer patients and doctors a more intuitive and diverse examination modality than traditional devices, with significantly higher diagnostic rates [33,34,35,36,37,38,75,76,77,78,79,80,115,116,117].

**The third trend** is the emergence of transmedia intelligent medical equipment. The “hand-eye system + doctor” is at the heart of the cross-media intelligent medical equipment. Overlaying and integrating images of lesions seen by doctors with images of previously examined lesions on the same platform allows doctors to more clearly compare changes in disease progression during follow-up visits [191,192].

**The fourth trend** is the medical devices and services + 5G network. The 5G network has three essential features. The first is broadband transmission, which facilitates the high-resolution remote transmission of medical images. The second is massive access and a quasi-equal clock, facilitating the remote control and remote observation of medical devices. The third is a high reliability, low-latency signal transmission. These three features have great supporting significance for the combination of medical devices with 5G, thus enabling the extensive and regular realization of telemedicine and promoting the formation of a new generation of medical devices [193,194,195,196,197].

**The fifth trend** is the widespread implementation of an intelligent cloud healthcare model based on the Internet of things (IoT). A new healthcare model has emerged supported by the intelligent cloud platform. The technology model combines smart devices with cloud platforms on the basis of IoT, which can not only be utilized by healthcare providers but also by patients and their families, and can connect another ancillary agency to the cloud platform for medical research, teaching and management. The models in healthcare are of great use in monitoring the progression of chronic skin diseases, as well as preventing and controlling them, and, through remote monitoring, hospitals and health-related institutions can access patient data and can further analyze them [198,199,200,201,202].

**The sixth trend** is the popularization of AI consultation in dermatology. In addition to intelligent recognition, AI can also perform consultations. There are already APPs and websites for automatic diabetes consultation that can answer common questions from patients with a single disease by giving a list of standardized questions and answers [203,204]. These initial consultations and interactions by AI can replace a certain amount of the doctor’s work and greatly improve the efficiency. For people in remote areas and those with limited access to healthcare, AI consultations would provide medical suggestions and direct guidance, thereby effectively delivering real-time help in an interactive format [205].

In conclusion, AI is unlikely to replace dermatologists at the moment, either on a technical level or on an ethical and legal level. AI still lacks some basic human qualities, such as compassion and human concern, which means that physicians should continue to assume their role here as the future link to the patient. In the future, AI should be the right hand of doctors, which can bring convenience to doctors and better services to patients. Over-promoting or avoiding AI is the incorrect attitude. Only with a proper understanding of AI can AI develop sustainably and bring help to dermatology.

## 9. The Challenge Posed to Humanity by the Development of AI

At present, there are several major challenges with the application of AI in the medical field: (1) The challenge of AI to the human way of thinking. The human doctors’ mind relies on common sense and, in many cases, personal preferences and emotions, whereas AI relies on historical judgement data to make correct or incorrect assertions [206]. With the introduction of deep learning, AI can highly mimic the way humans think and rely on neural networks for unsupervised self-learning. This quality can enable machines to learn far faster than humans and win several times against them [207]. As AI evolves, human doctors have a risk of being highly dependent on AI-assisted diagnostic systems and losing their enthusiasm for learning and self-improvement. (2) The threat to medical practitioner positions. Although dermatologists will not be replaced by AI at the moment, with the increasing amount of human knowledge and skills that are being acquired and surpassed by AI, more practitioners, such as medical technicians and nursing staff, could be replaced [208]. Whereas the first three industrial revolutions replaced human physical labor with machines, artificial intelligence not only replaces human physical labor, but also replaces a portion of mental labor [209]. This revolution has inevitably led to a dramatic transformation of the labor market. How to avoid the impact of AI development on employment is a considerable challenge. (3) The greatest prerequisite for the broad application of any AI is safety and security [210]. The greatest challenge is that humans lose control of AI or the novel technology controlled by non-humanitarians. If artificial intelligence loses control, the damage to humanity can be immeasurable. Artificial intelligence is built on algorithms, neural networks and large amounts of data [42,211]. The development of the internet and big data has made the security of artificial intelligence unpredictable. On the one hand, AI benefits from the Internet’s big data development resource advantage; on the other hand, the Internet’s human factor of hackers and viruses can pose a huge threat to AI [212]. Therefore, in the process of promoting AI-assisted diagnostic platforms and AI surgical robots, humans need to keep an eye on the uncontrollable consequences that it could potentially bring. (4) The new ethical and moral issues raised by AI are a common challenge for all doctors [213,214]. On the one hand, artificial intelligence can be a great facilitator for the treatment of dermatology. On the other hand, it can also have a huge impact on the current diagnosis and treatment patterns of skin diseases and surgeries. It is therefore of great concern to dermatologists and ethicists [215,216].

## 10. Prospects of the Application of AI in the Field of Dermatology

According to the summary above, we can see that AI for skin diseases represented by image recognition and analysis has now developed to a very advanced level. However, image recognition is only a part of clinical diagnosis and treatment, and medical service is a personalized service combining science and human care [217]. With the modification and refinement of AI technology and its closer integration with medical needs and scenarios, AI is expected to take on a part of boring and repetitive tasks, as well as improve the work efficiency of physicians, and is expected to alleviate the shortage of doctors [188,218]. AI can improve the accuracy of diagnosis and treatment, promote the optimal allocation of high-quality medical resources and push forward the efficient operation of a hierarchical medical system so as to accelerate the formation of medical consortia. For patients, it can provide large-scale quantitative analysis, promoting a more advanced stage of quantitative analysis in medical diagnosis, and spawn new diagnostic methods and treatment plans [174,219,220].

Healthcare is one of the industries that is most vulnerable to the impact of AI [221,222]. Whereas dermatologists have innovative, aesthetic, social and consultative strengths in healthcare, AI is unlikely to replace them both on a technical, ethical and legal level [208]. However, every day, dermatologists are also faced with a great deal of repetitive labor that does not require complex thinking and can be mastered through training. In the future, AI should be the right hand of doctors, which can bring convenience to doctors and better services to patients. Over-promoting or avoiding AI is the incorrect attitude. Only with a proper understanding of AI can AI develop sustainably and bring help to dermatology.

## 11. Conclusions and Perspective

This article demonstrates the enormous potential of AI-based diagnosis and assessment in dermatology-related fields. Besides the already established discrimination between nevus and melanoma, there are also many potential utilizations regarding diagnosing inflammatory dermatoses, evaluating skin beauty and assisting in dermatologic surgery. The quality and informative value of research data could be increased by using AI to improve their objectivity and reproducibility. AI can provide more detailed and precise suggestions for beauty consultation and improve the accuracy and efficiency of skin lesion diagnosis, as well as relieve doctors’ burden in daily work by taking over the drudgery. Although it is foreseeable that AI will outperform humans in certain well- defined decision-making areas, human interactions and human–AI symbiosis will remain indispensable in everyday clinical practice. The aim of applying AI is not to replace the dermatologist, but to expand their possibilities and approaches with a meaningful new tool. The use of AI in dermatology within the framework of human-AI symbiosis has proven to be crucial. While AI cannot achieve a 100% correct diagnosis rate, combining machines with physicians reliably enhances the system performance. It is conceivable that the AI-based procedures will be part of the daily routine of dermatologists.

## Figures and Tables

**Table 1:** Essential terminologies involved in AI in dermatology.

Terminology	Paraphrase
Artificial Intelligence (AI)	The intelligence manifested by machines made by humans, i.e., the ability of the machine to simulate natural intelligence.
Knowledge Representation	It is the field of AI dedicated to representing information about the world in a form that a computer system can utilize to solve complex tasks such as diagnosing a medical condition or having a dialog in a natural language.
Representation Learning (Feature Learning)	A set of techniques that allows a system to automatically discover the representations needed for feature detection or classification from raw data.
Machine Learning	The study of computer algorithms that improve automatically through experience. The algorithms use computational methods to learn from data without being explicitly programmed.
Deep Learning	A branch of machine learning methods based on artificial neural networks with representation learning.
Supervised Learning	Refers to the machine learning task of learning a function that maps an input to an output based on example input–output pairs. It infers a function from labeled training data consisting of a set of training examples.
Transfer Learning	Transfer learning is a machine learning model that allows a model developed from one task to be transferred for another task after fine-tuning and augmentation.
Artificial Neural Networks (ANNs)	ANNs, usually simply called neural networks (NNs), are computing systems vaguely inspired by the biological neural networks that constitute animal brains. An ANN is based on a collection of connected units or nodes called artificial neurons, which loosely model the neurons in a biological brain
Convolutional Neural Networks (CNNs)	CNNs are a class of neural networks; they are feedforward neural networks. Their artificial neurons can respond to a part of the surrounding units in the coverage area, most commonly applied to analyzing visual imagery.
Generative Adversarial Networks (GANs)	GANs are a method of unsupervised learning that learn by playing two neural networks against each other.
Pattern Recognition	The automated recognition of patterns and regularities in data. The environment and objects are collectively referred to as patterns.
Image Set	An object stores information about an image data set or a collection of image data sets. It contains image descriptions, locations of images and the number of images in the collection.

**Table 2:** The application of AI in multi-classification for skin lesions.

Authors	Refer ence	Year	Country	AI Algorithm Model	The Purpose of AI Algorithm	Image (Datasets) Recourse	No. of Images in Datasets	Usage	Types of Images	Accuracy /Precision (%)	Sensitivity/Recall (%)	Specificity (%)
Kassem et al.	[23]	2020	Egypt	Deep CNNs (modified GoogleNet)	Classification of multiple skin lesions	ISIC 2016–2019	25,331	Multi-class (8)	Dermoscopy	94.92	79.8	97
Rezvantalab et al.	[52]	2018	Iran	Four deep learning convolutional neural networks (CNNs)	Investigating the ability of deep convolutional neural networks in classification of multiple skin lesion	HAM10000; PH2	10,135	Multi-class (8)	Dermoscopy	80.22–89.01	82.26–99.10	79.60–89.01
Gessert et al.	[53]	2018	German y	Ensemble of CNN	Diagnosis of multiple skin lesions	ISIC-2018, HAM10000	23,515	Multi-class (7)	Dermoscopy	85.1	93.1–97.6	N/A
Gessert et al.	[54]	2020	German y	Ensemble of multi-resolution CNN	Classification of multiple skin lesions	HAM10000, BCN20000, MSK,7- point,	47,049	Multi-class (8)	Dermoscopy	80.5–96	72.5–74.2	94–99.9
Haenssle et al.	[55]	2018	German y	Deep convolutional neural network (Google’s Inception v4 architecture)	Detection of melanoma and comparison of its performance with 58 dermatologists	ISIC archive, clinical images	>150,000	Multi-class (20)	Macroscopy and Dermoscopy	86	86.6–88.9	71.3–75.7
Haenssle et al.	[56]	2020	Multi- country	FotoFinder^®^ Moleanalyzer Pro	Classification of skin lesions and comparison of the performance of the AI model with 96 dermatologists	ISIC archive, clinical images	>150,000	Multi-class (25)	Macroscopy and Dermoscopy	84	95	76.7
Esteva et al.	[66]	2017	USA	Deep convolutional neural networks (GoogleNet Inception v3)	Classification of skin cancer and comparison of the performance of AI model with 21 dermatologists	Online repositories and clinical data from	129,450	Multi-class (2032)	Macroscopy and Dermoscopy	1: 72.1 ± 0.9; 2: 55.4 ± 1.7	N/A	N/A
Mahbod et al.	[67]	2020	Austria	Multi-scale multi-convolutional neural networks (MSM-CNNs)	Investigating the effect of image size for skin lesion classification	ISIC-2016, 2017, 2018 HAM10000	12,927	Multi-class (7)	Dermoscopy	96.3	N/A	N/A
Iqbal et al.	[71]	2020	China	Deep CNN	Classification of multiple skin lesion	ISIC-2017, 2018, 2019	25,331	Multi-class (8)	Dermoscopy	94	93	91
Qin et al.	[73]	2020	China	Generative adversarial networks (GANs)	Classification of multiple skin lesion	ISIC-2018	10,015	Multi-class (7)	Dermoscopy	95.2	83.2	74.3
Cano et al.	[74]	2021	Panama	NasNet	Classification of multiple skin lesions	ISIC-2019	25,331	Multi-class (8)	Dermoscopy	71–99	73–98	70–99
Barhoumi et al.	[75]	2021	Tunisia	Transfer learning CNN model	Classification of multiple skin lesions	ISIC 2018	5057	Multi-class (7)	Dermoscopy	95	96	N/A
Ratul et al.	[76]	2020	Canada	Dilated CNNs (VGG-16,-19, MobileNet, Inception-V3)	Classification of multiple skin lesions	HAM10000	10,015	Multi-class (7)	Dermoscopy	87–89	87–89	N/A
Rashid et al.	[77]	2020	Pakistan	Semi-supervised GANs	Classification of multiple skin lesions	ISIC 2018	10,000	Multi-class (7)	Dermoscopy	73–94	69–92	N/A
Maron et al.	[78]	2019	German y	CNNs	Classification of multiple skin lesions and comparison of the performance of the AI model with 112 dermatologists	ISIC 2018, HAM10000	11,444	Multi-class (5)	Dermoscopy	N/A	90.2–97.7	94.2–99.5
Sun et al.	[79]	2021	China	CNNs	Classification of multiple skin lesions	ISIC-2019, MED- NODE, PH2, 7- point	18,460	Multi-class (7)	Dermoscopy	66.2–89.5	66.2–89.5	95.2–99.3
Jain et al.	[80]	2021	India	Six transfer learning nets	Classification of multiple skin lesions	HAM10000	10,015	Multi-class (7)	Dermoscopy	66–90	66–90	N/A
Winkler et al.	[81]	2020	Gemany	FotoFinder^®^ Moleanalyzer Pro (CNN)	Detection of various melanoma localizations and subtypes	ISIC archive, clinical images	>150,000	Multi-class (6)	Macroscopy and Dermoscopy	50.8–95.4	53.3–100	65–94
Binder et al.	[82]	1994	Austria	Artificial neural networks (ANNs)	Classification of naevi and malignant melanoma and comparison of the performance of AI model with 3 dermatologists	Oil immersion images of pigmented skin lesions	200	Multi-class (3)	Microscopy	86	95	88
Sies et al.	[83]	2020	German y	FotoFinder^®^ Moleanalyzer Pro/FotoFinder^®^Moleanalyzer- 3, Dynamole	Detection of various melanoma localizations and subtypes	ISIC dermoscopic archive, multicentric clinical images	>150,000	Multi-class (20)	Dermoscopy	92.8	77.6	95.3
Yang et al.	[84]	2020	China	CNNs (DenseNet-96, ResNet-152, ResNet-99)	Classification of multiple benign hyperpigmented dermatitis and comparison of the performance of AI model with 11 dermatologists	Clinical images	12,816	Multi-class (6)	Macroscopy	75.3–97.8	75.5–94.4	95.6–99.8
Lyakhov et al.	[85]	2022	Russia	Multimodal neural network	Recognition of multiple pigmented skin lesions	ISIC-2016–2021	41,725	Multi-class (10)	Dermoscopy	83.6	N/A	N/A
Guzman et al.	[86]	2015	Philippin es	Singe/multi-level and multi-models ANN	Detection of eczema skin lesion	Clinical images	504	Multi-class (3)	Macroscopy	Single: 78.17–87.30 Multi: 81.34–85.71	N/A	N/A
Han et al.	[87]	2018	Korea	Region-based convolutional deep neural networks	Diagnosis of onychomycosis and comparison of the performance of AI model with 42 dermatologists	Clinical images	49,567	Multi-class (6)	Macroscopy	82–98	82.7–96	69.3–96.7
A.Blum et al.	[88]	2004	Gemany	Vision algebra algorithms	Diagnosis of melanocytic lesions and validation of its diagnostic accuracy	Clinical images	837	Multi-class (20)	Dermoscopy	82.3–84.1	80–88.1	82.4–82.7
Marchetti et al.	[89]	2020	USA	CNNs and deep learning algorithms	Classification of melanoma and comparison of the performance of AI model with 17 dermatologists	ISIC-2017	2750	Multi-class (3)	Dermoscopy	86.8	76	85
Shen et al.	[90]	2018	China	Convolutional neural networks	Diagnosing for facial acne vulgaris	Clinical images	Binary: 6000 Multi:42,000	Binary-class/Multi-class (7)	Macroscopy	88.7–89.5	81.7–92	87–95.7
Seité et al.	[91]	2019	France	Deep learning algorithm	Determination of the severity of facial acne and identification of subtypes of acne lesion	Clinical images	4958	Multi-class (3)	Macroscopy	N/A	N/A	N/A
Zhao et al.	[92]	2019	China	CNNs	Identification of psoriasis	XiangyaDerm-Pso9	8021	Multi-class (9)	Macroscopy	88	83–95	96–98
Han et al.	[93]	2020	Korea	Deep Neural Networks	Predicting malignancy and suggesting treatment option, as well as multi-classification for 134 skin disorders	Clinical images	220,680	1:Binary- class 2:Multi-class (134)	Macroscopy	1: 56.7–92 2: 44.8–78.1	N/A	N/A

**Table 3:** The application of AI in binary classification for skin lesions.

Authors	Reference	Year	Country	AI Algorithm Model	The Purpose of AI Algorithm	Image (Datasets) Recourse	No. of Images in Datasets	Types of Images	Accuracy/ Precision (%)	Sensitivity/Recall (%)	Specificity (%)
Filho et al.	[51]	2018	Germany	Structural Co-occurrence matrix	Classification of melanoma	ISIC-2016, 2017, PH2	3100	Dermoscopy	89.93–99	89.9–99.2	95.15–99.4
Marchetti et al.	[89]	2018	USA	Non-learned approaches and machine learning methods	Classification of melanoma and comparison of the performance of AI model with 8 dermatologists	ISIC-2016	1279	Dermoscopy	85–86	46–70	88–92
Roffman et al.	[94]	2018	USA	Artificial neural network	Detection of non-melanoma skin cancer	NHIS 1997–2015	462,630	Macroscopy	81	86.2–88.5	62.2–62.7
Alzubaidi	[95]	2021	Australia	Transfer learning model	Discrimination of skin cancer and normal skin	ISIC-2016–2020, Med- Node, Dermofit	>200,000	Dermoscopy	89.69–98.57	85.60–97.90	N/A
Guimarães et al.	[96]	2020	Germany	Convolutional neural networks	Diagnosis of atopic dermatitis	Multiphoton tomography Images	3663	Multiphoton tomograph	97.0 ± 0.2	96.6 ± 0.2	97.7 ± 0.3
Ho. et al.	[97]	2020	USA	Deep neural network	Image segmentation of plexiform neurofibromas	MRI images	35	MRI	N/A	N/A	N/A
Fink et al.	[98]	2018	Germany	Edge-preserving thresholding automated shape recognition	Classification of psoriasis and measurement of lesion area andseverity index	Clinical images	10 patients	Macroscopy	N/A	N/A	N/A
Fink et al.	[99]	2019	Germany	Edge-preserving thresholding automated shape recognition	Validation of the precision and reproducibility of algorithm in PASI measurements	Clinical images	120 patients	Macroscopy	N/A	N/A	N/A
Schnuerle et al.	[100]	2017	Switzerland	Support vector machines	Detection of hand eczema	Clinical images	N/A	Macroscopy	74.5–89.29	48–71.43	77.24–93.63
Gao et al.	[101]	2020	Chinas	Deep learning network architecture (ResNet-50)	Detection for fungal skin lesion	Clinical images	292	Macroscopy	N/A	95.2–99.5	91.4–100
Bashat et al.	[102]	2018	Israel	N/A	Differentiation of benign and malignant neurofibroma	MRI images	30	MRI	80	72	87
Duarte et al.	[103]	2014	Portugal	Support vector machines	Classification of whole-brain grey and white matter of MRI between NF1 patients and normal person	T1-weighted MRI scans	99	MRI Images	94	92	96
Meienberger et al.	[104]	2019	Switzerland	Convolutional neural networks (Net 16)	Establishment of an accurate and objective psoriasis assessment method	Clinical images	203	Macroscopy	92	N/A	N/A
Gustafson et al.	[105]	2017	USA	Electronic health record based phenotype algorithm	Identification of atopic dermatitis and comparison of the performance of AI model with 4 dermatologists	Clinical images	562	N/A	N/A	53.6–75	N/A
Luo et al.	[106]	2020	China	Cycle-consistent adversarial networks	Classification of vitiligo skin lesion	Clinical Images	80,000	Macroscopy	85.69	80.73	66.2
Makena et al.	[107]	2019	USA	Convolutional neural networks	Segmentation of vitiligo skin lesion	RGB images of vitiligo lesions	308	Macroscopy (UV/natural light)	74–88.7	N/A	N/A

**Table 4:** The application of AI in aesthetic dermatology.

Authors	Reference	Year	Country	AI Algorithm Model	The Purpose of AI Algorithm	Image (Datasets) Recourse	No. of Images in Datasets	Types of Images	Accuracy/Precision (%)
Eisentha et al.	[64]	2006	Israel	Deep learning algorithm	Predicting facial attractiveness ratings	Volunteer images	194	Macroscopy	0.65 correlation with human
Kagian et al.	[65]	2008	Israel	Linear regression algorithm	Extraction of facial features from raw images and rating facial attractiveness	Volunteer images	91	Macroscopy	0.82 correlation with human
Zhang et al.	[108]	2017	China	Hypergraph-based semi-supervised learning method (HSSL)	Analysis of human face attractiveness	Shanghai Database and celebrity portrait from Internet	2354	Macroscopy	81.47–84.21

## Data Availability

Not applicable.

## References

[1] Bellman R. (1978). An Introduction to Artificial Intelligence: Can Computers Think?.

[2] Litjens G., Kooi T., Bejnordi B.E., Setio A.A.A., Ciompi F., Ghafoorian M., van der Laak J.A.W.M., van Ginneken B., Sánchez C.I. (2017). A Survey on Deep Learning in Medical Image Analysis. Med. Image Anal..

[3] Laino M.E., Cancian P., Politi L.S., Della Porta M.G., Saba L., Savevski V. (2022). Generative Adversarial Networks in Brain Imaging: A Narrative Review. J. Imaging.

[4] Lassau N., Bousaid I., Chouzenoux E., Lamarque J.P., Charmettant B., Azoulay M., Cotton F., Khalil A., Lucidarme O., Pigneur F. (2020). Three Artificial Intelligence Data Challenges Based on CT and MRI. Diagn. Interv. Imaging.

[5] Wang S., Celebi M.E., Zhang Y.D., Yu X., Lu S., Yao X., Zhou Q., Miguel M.G., Tian Y., Gorriz J.M. (2021). Advances in Data Preprocessing for Bio-Medical Data Fusion: An Overview of the Methods, Challenges, and Prospects. Inf. Fusion.

[6] von Itzstein M.S., Hullings M., Mayo H., Beg M.S., Williams E.L., Gerber D.E. (2021). Application of Information Technology to Clinical Trial Evaluation and Enrollment: A Review. JAMA Oncol..

[7] Xu Y., Su G.-H., Ma D., Xiao Y., Shao Z.-M., Jiang Y.-Z. (2021). Technological Advances in Cancer Immunity: From Immunogenomics to Single-Cell Analysis and Artificial Intelligence. Signal Transduct. Target. Ther..

[8] Barisoni L., Lafata K.J., Hewitt S.M., Madabhushi A., Balis U.G.J. (2020). Digital Pathology and Computational Image Analysis in Nephropathology. Nat. Rev. Nephrol..

[9] Ho D., Quake S.R., McCabe E.R.B., Chng W.J., Chow E.K., Ding X., Gelb B.D., Ginsburg G.S., Hassenstab J., Ho C.-M. (2020). Enabling Technologies for Personalized and Precision Medicine. Trends Biotechnol..

[10] Bajaj S., Marchetti M.A., Navarrete-Dechent C., Dusza S.W., Kose K., Marghoob A.A. (2016). The Role of Color and Morphologic Characteristics in Dermoscopic Diagnosis. JAMA Dermatol..

[11] Perednia D.A. (1991). What Dermatologists Should Know about Digital Imaging. J. Am. Acad. Dermatol..

[12] Møllersen K., Kirchesch H., Zortea M., Schopf T.R., Hindberg K., Godtliebsen F. (2015). Computer-Aided Decision Support for Melanoma Detection Applied on Melanocytic and Nonmelanocytic Skin Lesions: A Comparison of Two Systems Based on Automatic Analysis of Dermoscopic Images. BioMed Res. Int..

[13] Cazzaniga S., Sassi F., Mercuri S.R., Naldi L. (2009). Prediction of Clinical Response to Excimer Laser Treatment in Vitiligo by Using Neural Network Models. Dermatology.

[14] Khozeimeh F., Alizadehsani R., Roshanzamir M., Khosravi A., Layegh P., Nahavandi S. (2017). An Expert System for Selecting Wart Treatment Method. Comput. Biol. Med..

[15] Kim S.I., Lee S., Jeong C.W., Kim H.S. (2018). Robot-Assisted Anterior Pelvic Exenteration in Vulvovaginal Malignant Melanoma. Gynecol. Oncol..

[16] Rose P.T., Nusbaum B. (2014). Robotic Hair Restoration. Dermatol. Clin..

[17] Du-Harpur X., Watt F.M., Luscombe N.M., Lynch M.D. (2020). What Is AI? Applications of Artificial Intelligence to Dermatology. Br. J. Dermatol..

[18] Young A.T., Xiong M., Pfau J., Keiser M.J., Wei M.L. (2020). Artificial Intelligence in Dermatology: A Primer. J. Investig. Dermatol..

[19] Hogarty D.T., Su J.C., Phan K., Attia M., Hossny M., Nahavandi S., Lenane P., Moloney F.J., Yazdabadi A. (2020). Artificial Intelligence in Dermatology—Where We Are and the Way to the Future: A Review. Am. J. Clin. Dermatol..

[20] Codella N.C.F., Gutman D., Celebi M.E., Helba B., Marchetti M.A., Dusza S.W., Kalloo A., Liopyris K., Mishra N., Kittler H. Skin Lesion Analysis toward Melanoma Detection: A Challenge at the 2017 International Symposium on Biomedical Imaging (ISBI), Hosted by the International Skin Imaging Collaboration (ISIC). Proceedings of the 2018 IEEE 15th International Symposium on Biomedical Imaging (ISBI 2018).

[21] Codella N., Rotemberg V., Tschandl P., Celebi M.E., Dusza S., Gutman D., Helba B., Kalloo A., Liopyris K., Marchetti M. Skin Lesion Analysis Toward Melanoma Detection 2018: A Challenge Hosted by the International Skin Imaging Collaboration (ISIC). Proceedings of the 2018 IEEE 15th International Symposium on Biomedical Imaging (ISBI 2018).

[22] Marchetti M.A., Liopyris K., Dusza S.W., Codella N.C.F., Gutman D.A., Helba B., Kalloo A., Halpern A.C. (2020). International Skin Imaging Collaboration. Computer algorithms show potential for improving dermatologists’ accuracy to diagnose cutaneous melanoma: Results of the International Skin Imaging Collaboration 2017. J. Am. Acad. Dermatol..

[23] Kassem M.A., Hosny K.M., Fouad M.M. (2020). Skin Lesions Classification into Eight Classes for ISIC 2019 Using Deep Convolutional Neural Network and Transfer Learning. IEEE Access.

[24] Gottfredson L.S. (1994). Mainstream Science on Intelligence: An Editorial With 52 Signatories, History, and Bibliography. Intelligence.

[25] Russell S., Norvig P. (2020). Artificial Intelligence: A Modern Approach.

[26] Monett D., Lewis C.W.P., Thórisson K.R., Bach J., Baldassarre G., Granato G., Berkeley I.S.N., Chollet F., Crosby M., Shevlin H. (2020). Special Issue “On Defining Artificial Intelligence”—CommentarJohn McCarthy’s Definition of Intelligence.Ies and Author’s Response. J. Artif. Gen. Intell..

[27] Kaplan A., Haenlein M. (2019). Siri, Siri, in My Hand: Who’s the Fairest in the Land?. On the Interpretations, Illustrations, and Implications of Artificial Intelligence. Bus. Horiz..

[28] Turing A.M. (1936). On Computable Numbers, with an Application to the Entscheidungsproblem. J. Math..

[29] McCulloch W.S., Pitts W. (1943). A Logical Calculus of the Ideas Immanent in Nervous Activity. Bull. Math. Biophys..

[30] McCorduck P. (2004). Machines Who Think: A Personal Inquiry into the History and Prospects of Artificial Intelligence.

[31] Widrow B., Lehr M.A. (1990). 30 Years of Adaptive Neural Networks: Perceptron, Madaline, and Backpropagation. Proc. IEEE.

[32] Lodwick G.S., Keats T.E., Dorst J.P. (1963). The Coding of Roentgen Images for Computer Analysis as Applied to Lung Cancer. Radiology.

[33] Lindsay R.K., Buchanan B.G., Feigenbaum E.A., Lederberg J. (1980). Applications of Artificial Intelligence for Organic Chemistry: The DENDRAL Project.

[34] Gao Y., Geras K.J., Lewin A.A., Moy L. (2019). New Frontiers: An Update on Computer-Aided Diagnosis for Breast Imaging in the Age of Artificial Intelligence. Am. J. Roentgenol..

[35] Gutkowicz-Krusin D., Elbaum M., Jacobs A., Keem S., Kopf A.W., Kamino H., Wang S., Rubin P., Rabinovitz H., Oliviero M. (2000). Precision of Automatic Measurements of Pigmented Skin Lesion Parameters with a MelaFindTM Multispectral Digital Dermoscope. Melanoma Res..

[36] Esteva A., Robicquet A., Ramsundar B., Kuleshov V., DePristo M., Chou K., Cui C., Corrado G., Thrun S., Dean J. (2019). A Guide to Deep Learning in Healthcare. Nat. Med..

[37] Jumper J., Evans R., Pritzel A., Green T., Figurnov M., Ronneberger O., Tunyasuvunakool K., Bates R., Žídek A., Potapenko A. (2021). Highly Accurate Protein Structure Prediction with AlphaFold. Nature.

[38] Karras T., Aittala M., Laine S., Härkönen E., Hellsten J., Lehtinen J., Aila T. (2021). Alias-Free Generative Adversarial Networks. Adv. Neural Inf. Process. Syst..

[39] Goyal P., Caron M., Lefaudeux B., Xu M., Wang P., Pai V., Singh M., Liptchinsky V., Misra I., Joulin A. (2021). Self-Supervised Pretraining of Visual Features in the Wild. arXiv.

[40] Russell S.J., Norvig P. (2007). Artificial Intelligence. The ACM Computing Classification System 1998.

[41] Mitchell T. (1997). Machine Learning.

[42] Yegnanarayana B. (2004). Artificial Neural Networks.

[43] Erickson B.J., Korfiatis P., Kline T.L., Akkus Z., Philbrick K., Weston A.D. (2018). Deep Learning in Radiology: Does One Size Fit All?. J. Am. Coll. Radiol..

[44] Manne R., Kantheti S., Kantheti S. (2020). Classification of Skin Cancer Using Deep Learning, Convolutional Neural Networks—Opportunities and Vulnerabilities—A Systematic Review. Int. J. Mod. Trends Sci. Technol..

[45] Dildar M., Akram S., Irfan M., Khan H.U., Ramzan M., Mahmood A.R., Alsaiari S.A., Saeed A.H.M., Alraddadi M.O., Mahnashi M.H. (2021). Skin Cancer Detection: A Review Using Deep Learning Techniques. Int. J. Environ. Res. Public Health.

[46] Fink C., Blum A., Buhl T., Mitteldorf C., Hofmann-Wellenhof R., Deinlein T., Stolz W., Trennheuser L., Cussigh C., Deltgen D. (2020). Diagnostic Performance of a Deep Learning Convolutional Neural Network in the Differentiation of Combined Naevi and Melanomas. J. Eur. Acad. Dermatol. Venereol..

[47] Alzubaidi L., Zhang J., Humaidi A.J., Al-Dujaili A., Duan Y., Al-Shamma O., Santamaría J., Fadhel M.A., Al-Amidie M., Farhan L. (2021). Review of Deep Learning: Concepts, CNN Architectures, Challenges, Applications, Future Directions.

[48] Ghosh A., Sufian A., Sultana F., Chakrabarti A., De D. (2020). Fundamental Concepts of Convolutional Neural Network. Recent Trends and Advances in Artificial Intelligence and Internet of Things.

[49] Daneshjou R., Vodrahalli K., Novoa R.A., Jenkins M., Liang W., Rotemberg V., Ko J., Swetter S.M., Bailey E.E., Gevaert O. (2022). Disparities in Dermatology AI Performance on a Diverse, Curated Clinical Image Set. arXiv.

[50] Zhao Z.-Q., Xu S.-T., Liu D., Tian W.-D., Jiang Z.-D. (2019). A Review of Image Set Classification. Neurocomputing.

[51] Rebouças Filho P.P., Peixoto S.A., Medeiros da Nóbrega R.V., Hemanth D.J., Medeiros A.G., Sangaiah A.K., de Albuquerque V.H.C. (2018). Automatic Histologically-Closer Classification of Skin Lesions. Comput. Med. Imaging Graph..

[52] Rezvantalab A., Safigholi H., Karimijeshni S. (2018). Dermatologist Level Dermoscopy Skin Cancer Classification Using Different Deep Learning Convolutional Neural Networks Algorithms. arXiv.

[53] Gessert N., Sentker T., Madesta F., Schmitz R., Kniep H., Baltruschat I., Werner R., Schlaefer A. (2018). Skin Lesion Diagnosis Using Ensembles, Unscaled Multi-Crop Evaluation and Loss Weighting. arXiv.

[54] Gessert N., Nielsen M., Shaikh M., Werner R., Schlaefer A. (2020). Skin Lesion Classification Using Ensembles of Multi-Resolution EfficientNets with Meta Data. MethodsX.

[55] Haenssle H.A., Fink C., Schneiderbauer R., Toberer F., Buhl T., Blum A., Kalloo A., Ben Hadj Hassen A., Thomas L., Enk A. (2018). Man against Machine: Diagnostic Performance of a Deep Learning Convolutional Neural Network for Dermoscopic Melanoma Recognition in Comparison to 58 Dermatologists. Ann. Oncol..

[56] Haenssle H.A., Fink C., Toberer F., Winkler J., Stolz W., Deinlein T., Hofmann-Wellenhof R., Lallas A., Emmert S., Buhl T. (2020). Man against Machine Reloaded: Performance of a Market-Approved Convolutional Neural Network in Classifying a Broad Spectrum of Skin Lesions in Comparison with 96 Dermatologists Working under Less Artificial Conditions. Ann. Oncol..

[57] Berk-Krauss J., Polsky D., Stein J.A. (2017). Mole Mapping for Management of Pigmented Skin Lesions. Dermatol. Clin..

[58] Demers A.A., Nugent Z., Mihalcioiu C., Wiseman M.C., Kliewer E.V. (2005). Trends of Nonmelanoma Skin Cancer from 1960 through 2000 in a Canadian Population. J. Am. Acad. Dermatol..

[59] Weinberg J., Kaddu S., Gabler G., Kovarik C. (2009). The African Teledermatology Project: Providing Access to Dermatologic Care and Education in Sub-Saharan Africa. Pan Afr. Med. J..

[60] Gaffney R., Rao B. (2015). Global Teledermatology. Glob. Dermatol..

[61] Kaliyadan F., Ashique K.T. (2020). Use of Mobile Applications in Dermatology. Indian J. Dermatol..

[62] Freeman K., Dinnes J., Chuchu N., Takwoingi Y., Bayliss S.E., Matin R.N., Jain A., Walter F.M., Williams H.C., Deeks J.J. (2020). Algorithm Based Smartphone Apps to Assess Risk of Skin Cancer in Adults: Systematic Review of Diagnostic Accuracy Studies. BMJ.

[63] Veronese F., Branciforti F., Zavattaro E., Tarantino V., Romano V., Meiburger K.M., Salvi M., Seoni S., Savoia P. (2021). The Role in Teledermoscopy of an Inexpensive and Easy-to-Use Smartphone Device for the Classification of Three Types of Skin Lesions Using Convolutional Neural Networks. Diagnostics.

[64] Eisentha Y., Dror G., Ruppin E. (2006). Facial Attractiveness: Beauty and the Machine. Neural Comput..

[65] Kagian A., Dror G., Leyvand T., Meilijson I., Cohen-Or D., Ruppin E. (2008). A Machine Learning Predictor of Facial Attractiveness Revealing Human-like Psychophysical Biases. Vis. Res..

[66] Esteva A., Kuprel B., Novoa R.A., Ko J., Swetter S.M., Blau H.M., Thrun S. (2017). Dermatologist-Level Classification of Skin Cancer with Deep Neural Networks. Nature.

[67] Mahbod A., Tschandl P., Langs G., Ecker R., Ellinger I. (2020). The Effects of Skin Lesion Segmentation on the Performance of Dermatoscopic Image Classification. Comput. Methods Programs Biomed..

[68] Mahbod A., Schaefer G., Wang C., Dorffner G., Ecker R., Ellinger I. (2020). Transfer Learning Using a Multi-Scale and Multi-Network Ensemble for Skin Lesion Classification. Comput. Methods Programs Biomed..

[69] Mirikharaji Z., Abhishek K., Izadi S., Hamarneh G. D-LEMA: Deep Learning Ensembles from Multiple Annotations-Application to Skin Lesion Segmentation. Proceedings of the 2021 IEEE/CVF Conference on Computer Vision and Pattern Recognition Workshops (CVPRW).

[70] Yang C.-H., Ren J.-H., Huang H.-C., Chuang L.-Y., Chang P.-Y. (2021). Deep Hybrid Convolutional Neural Network for Segmentation of Melanoma Skin Lesion. Comput. Intell. Neurosci..

[71] Iqbal I., Younus M., Walayat K., Kakar M.U., Ma J. (2021). Automated Multi-Class Classification of Skin Lesions through Deep Convolutional Neural Network with Dermoscopic Images. Comput. Med. Imaging Graph..

[72] Mirikharaji Z., Yan Y., Hamarneh H. (2019). Learning to Segment Skin Lesions from Noisy Annotations.

[73] Qin Z., Liu Z., Zhu P., Xue Y. (2020). A GAN-Based Image Synthesis Method for Skin Lesion Classification. Comput. Methods Programs Biomed..

[74] Cano E., Mendoza-Avilés J., Areiza M., Guerra N., Mendoza-Valdés J.L., Rovetto C.A. (2021). Multi Skin Lesions Classification Using Fine-Tuning and Data-Augmentation Applying Nasnet. PeerJ Comput. Sci..

[75] Barhoumi W., Khelifa A. (2021). Skin Lesion Image Retrieval Using Transfer Learning-Based Approach for Query-Driven Distance Recommendation. Comput. Biol. Med..

[76] Ratul M.A.R., Mozaffari M.H., Lee W.-S., Parimbelli E. (2020). Skin Lesions Classification Using Deep Learning Based on Dilated Convolution. bioRxiv.

[77] Rashid H., Tanveer M.A., Aqeel Khan H. Skin Lesion Classification Using GAN Based Data Augmentation. Proceedings of the 2019 41st Annual International Conference of the IEEE Engineering in Medicine and Biology Society (EMBC).

[78] Maron R.C., Weichenthal M., Utikal J.S., Hekler A., Berking C., Hauschild A., Enk A.H., Haferkamp S., Klode J., Schadendorf D. (2019). Systematic Outperformance of 112 Dermatologists in Multiclass Skin Cancer Image Classification by Convolutional Neural Networks. Eur. J. Cancer.

[79] Sun Q., Huang C., Chen M., Xu H., Yang Y. (2021). Skin Lesion Classification Using Additional Patient Information. BioMed Res. Int..

[80] Jain S., Singhania U., Tripathy B., Nasr E.A., Aboudaif M.K., Kamrani A.K. (2021). Deep Learning-Based Transfer Learning for Classification of Skin Cancer. Sensors.

[81] Winkler J.K., Sies K., Fink C., Toberer F., Enk A., Deinlein T., Hofmann-Wellenhof R., Thomas L., Lallas A., Blum A. (2020). Melanoma Recognition by a Deep Learning Convolutional Neural Network—Performance in Different Melanoma Subtypes and Localisations. Eur. J. Cancer.

[82] Binder M., Steiner A., Schwarz M., Knollmayer S., Wolff K., Pehamberger H. (1994). Application of an Artificial Neural Network in Epiluminescence Microscopy Pattern Analysis of Pigmented Skin Lesions: A Pilot Study. Br. J. Dermatol..

[83] Sies K., Winkler J.K., Fink C., Bardehle F., Toberer F., Buhl T., Enk A., Blum A., Rosenberger A., Haenssle H.A. (2020). Past and Present of Computer-Assisted Dermoscopic Diagnosis: Performance of a Conventional Image Analyser versus a Convolutional Neural Network in a Prospective Data Set of 1,981 Skin Lesions. Eur. J. Cancer.

[84] Yang Y., Ge Y., Guo L., Wu Q., Peng L., Zhang E., Xie J., Li Y., Lin T. (2021). Development and Validation of Two Artificial Intelligence Models for Diagnosing Benign, Pigmented Facial Skin Lesions. Ski. Res. Technol..

[85] Lyakhov P.A., Lyakhova U.A., Nagornov N.N. (2022). System for the Recognizing of Pigmented Skin Lesions with Fusion and Analysis of Heterogeneous Data Based on a Multimodal Neural Network. Cancers.

[86] De Guzman L.C., Maglaque R.P.C., Torres V.M.B., Zapido S.P.A., Cordel M.O. Design and Evaluation of a Multi-Model, Multi-Level Artificial Neural Network for Eczema Skin Lesion Detection. Proceedings of the 2015 3rd International Conference on Artificial Intelligence, Modelling and Simulation (AIMS).

[87] Han S.S., Park G.H., Lim W., Kim M.S., Na J.I., Park I., Chang S.E. (2018). Deep Neural Networks Show an Equivalent and Often Superior Performance to Dermatologists in Onychomycosis Diagnosis: Automatic Construction of Onychomycosis Datasets by Region-Based Convolutional Deep Neural Network. PLoS ONE.

[88] Blum A., Luedtke H., Ellwanger U., Schwabe R., Rassner G., Garbe C. (2004). Digital Image Analysis for Diagnosis of Cutaneous Melanoma. Development of a Highly Effective Computer Algorithm Based on Analysis of 837 Melanocytic Lesions. Br. J. Dermatol..

[89] Marchetti M.A., Codella N.C.F., Dusza S.W., Gutman D.A., Helba B., Kalloo A., Mishra N., Carrera C., Celebi M.E., DeFazio J.L. (2018). Results of the 2016 International Skin Imaging Collaboration International Symposium on Biomedical Imaging Challenge: Comparison of the Accuracy of Computer Algorithms to Dermatologists for the Diagnosis of Melanoma from Dermoscopic Images. J. Am. Acad. Dermatol..

[90] Shen X., Zhang J., Yan C., Zhou H. (2018). An Automatic Diagnosis Method of Facial Acne Vulgaris Based on Convolutional Neural Network. Sci. Rep..

[91] Seité S., Khammari A., Benzaquen M., Moyal D., Dréno B. (2019). Development and Accuracy of an Artificial Intelligence Algorithm for Acne Grading from Smartphone Photographs. Exp. Dermatol..

[92] Zhao S., Xie B., Li Y., Zhao X., Kuang Y., Su J., He X., Wu X., Fan W., Huang K. (2020). Smart Identification of Psoriasis by Images Using Convolutional Neural Networks: A Case Study in China. J. Eur. Acad. Dermatol. Venereol..

[93] Han S.S., Park I., Eun Chang S., Lim W., Kim M.S., Park G.H., Chae J.B., Huh C.H., Na J.I. (2020). Augmented Intelligence Dermatology: Deep Neural Networks Empower Medical Professionals in Diagnosing Skin Cancer and Predicting Treatment Options for 134 Skin Disorders. J. Investig. Dermatol..

[94] Roffman D., Hart G., Girardi M., Ko C.J., Deng J. (2018). Predicting Non-Melanoma Skin Cancer via a Multi-Parameterized Artificial Neural Network. Sci. Rep..

[95] Alzubaidi L., Al-Amidie M., Al-Asadi A., Humaidi A.J., Al-Shamma O., Fadhel M.A., Zhang J., Santamaría J., Duan Y. (2021). Novel Transfer Learning Approach for Medical Imaging with Limited Labeled Data. Cancers.

[96] Guimarães P., Batista A., Zieger M., Kaatz M., Koenig K. (2020). Artificial Intelligence in Multiphoton Tomography: Atopic Dermatitis Diagnosis. Sci. Rep..

[97] Ho C.Y., Kindler J.M., Persohn S., Kralik S.F., Robertson K.A., Territo P.R. (2020). Image Segmentation of Plexiform Neurofibromas from a Deep Neural Network Using Multiple B-Value Diffusion Data. Sci. Rep..

[98] Fink C., Fuchs T., Enk A., Haenssle H.A. (2018). Design of an Algorithm for Automated, Computer-Guided PASI Measurements by Digital Image Analysis. J. Med. Syst..

[99] Fink C., Alt C., Uhlmann L., Klose C., Enk A., Haenssle H.A. (2019). Precision and Reproducibility of Automated Computer-Guided Psoriasis Area and Severity Index Measurements in Comparison with Trained Physicians. Br. J. Dermatol..

[100] Schnürle S., Pouly M., Vor Der Brück T., Navarini A., Koller T. On Using Support Vector Machines for the Detection and Quantification of Hand Eczema. Proceedings of the 9th International Conference on Agents and Artificial Intelligence (ICAART 2017).

[101] Gao W., Li M., Wu R., Du W., Zhang S., Yin S., Chen Z., Huang H. (2021). The Design and Application of an Automated Microscope Developed Based on Deep Learning for Fungal Detection in Dermatology. Mycoses.

[102] Bashat D.B., Artzi M., Ganut T., Vitinshtein F., Ben-Sira L., Bokstein F. Differentiation between Plexiform Neurofibromas and Malignant Nerve Sheath Tumors in Patients with Neurofibromatosis Type 1 (NF1) Using Radiomics Analysis of MRI. Proceedings of the European Congress of Radiology-ECR 2019.

[103] Duarte J.V., Ribeiro M.J., Violante I.R., Cunha G., Silva E., Castelo-Branco M. (2014). Multivariate Pattern Analysis Reveals Subtle Brain Anomalies Relevant to the Cognitive Phenotype in Neurofibromatosis Type 1. Hum. Brain Mapp..

[104] Meienberger N., Anzengruber F., Amruthalingam L., Christen R., Koller T., Maul J.T., Pouly M., Djamei V., Navarini A.A. (2020). Observer-Independent Assessment of Psoriasis-Affected Area Using Machine Learning. J. Eur. Acad. Dermatol. Venereol..

[105] Gustafson E., Pacheco J., Wehbe F., Silverberg J., Thompson W. A Machine Learning Algorithm for Identifying Atopic Dermatitis in Adults from Electronic Health Records. Proceedings of the 2017 IEEE International Conference on Healthcare Informatics (ICHI).

[106] Luo W., Liu J., Huang Y., Zhao N. (2020). An Effective Vitiligo Intelligent Classification System. J. Ambient Intell. Humaniz. Comput..

[107] Low M., Huang V., Raina P. Automating Vitiligo Skin Lesion Segmentation Using Convolutional Neural Networks. Proceedings of the 2020 IEEE 17th International Symposium on Biomedical Imaging (ISBI).

[108] Zhang L., Zhang D., Sun M.M., Chen F.M. (2017). Facial Beauty Analysis Based on Geometric Feature: Toward Attractiveness Assessment Application. Expert Syst. Appl..

[109] Cassidy B., Kendrick C., Brodzicki A., Jaworek-Korjakowska J., Yap M.H. (2022). Analysis of the ISIC Image Datasets: Usage, Benchmarks and Recommendations. Med. Image Anal..

[110] Garcia-Arroyo J.L., Garcia-Zapirain B. (2019). Segmentation of Skin Lesions in Dermoscopy Images Using Fuzzy Classification of Pixels and Histogram Thresholding. Comput. Methods Programs Biomed..

[111] Lucius M., De All J., De All J.A., Belvisi M., Radizza L., Lanfranconi M., Lorenzatti V., Galmarini C.M. (2020). Deep Neural Frameworks Improve the Accuracy of General Practitioners in the Classification of Pigmented Skin Lesions. Diagnostics.

[112] Minagawa A., Koga H., Sano T., Matsunaga K., Teshima Y., Hamada A., Houjou Y., Okuyama R. (2021). Dermoscopic Diagnostic Performance of Japanese Dermatologists for Skin Tumors Differs by Patient Origin: A Deep Learning Convolutional Neural Network Closes the Gap. J. Dermatol..

[113] Al-masni M.A., Kim D.-H.H., Kim T.-S.S. (2020). Multiple Skin Lesions Diagnostics via Integrated Deep Convolutional Networks for Segmentation and Classification. Comput. Methods Programs Biomed..

[114] Singhal A., Shukla R., Kankar P.K., Dubey S., Singh S., Pachori R.B. (2020). Comparing the Capabilities of Transfer Learning Models to Detect Skin Lesion in Humans. Proc. Inst. Mech. Eng. Part H J. Eng. Med..

[115] Le D.N.T., Le H.X., Ngo L.T., Ngo H.T. (2020). Transfer Learning with Class-Weighted and Focal Loss Function for Automatic Skin Cancer Classification. arXiv.

[116] Lei B., Xia Z., Jiang F., Jiang X., Ge Z., Xu Y., Qin J., Chen S., Wang T., Wang S. (2020). Skin Lesion Segmentation via Generative Adversarial Networks with Dual Discriminators. Med. Image Anal..

[117] International Skin Imaging Collaboration (ISIC) Sixth ISIC Skin Image Analysis Workshop@ CVPR 2021 Virtual. https://workshop2021.isic-archive.com.

[118] Ferrara G., Argenziano G. (2021). The WHO 2018 Classification of Cutaneous Melanocytic Neoplasms: Suggestions from Routine Practice. Front. Oncol..

[119] Braun R.P., Rabinovitz H.S., Oliviero M., Kopf A.W., Saurat J.-H. (2005). Dermoscopy of Pigmented Skin Lesions. J. Am. Acad. Dermatol..

[120] Tschandl P., Codella N., Akay B.N., Argenziano G., Braun R.P., Cabo H., Gutman D., Halpern A., Helba B., Hofmann-wellenhof R. (2019). Comparison of the Accuracy of Human Readers versus Machine-Learning Algorithms for Pigmented Skin Lesion Classification: An Open, Web-Based, International, Diagnostic Study. Lancet Oncol..

[121] Penn L., Rothman L., Sutton A.M., Brinster N.K., Vidal C.I. (2019). What’s New in Dermatopathology: Inflammatory Dermatoses. Adv. Anat. Pathol..

[122] Ferlay J., Colombet M., Soerjomataram I., Mathers C., Parkin D.M., Piñeros M., Znaor A., Bray F. (2019). Estimating the Global Cancer Incidence and Mortality in 2018: GLOBOCAN Sources and Methods. Int. J. Cancer.

[123] Leiter U., Keim U., Garbe C., Reichrath J. (2020). Epidemiology of Skin Cancer: Update 2019. Sunlight, Vitamin D and Skin Cancer.

[124] Lomas A., Leonardi-Bee J., Bath-Hextall F. (2012). A Systematic Review of Worldwide Incidence of Nonmelanoma Skin Cancer. Br. J. Dermatol..

[125] Schadendorf D., van Akkooi A.C.J., Berking C., Griewank K.G., Gutzmer R., Hauschild A., Stang A., Roesch A., Ugurel S. (2018). Melanoma. Lancet.

[126] Sung H., Ferlay J., Siegel R.L., Laversanne M., Soerjomataram I., Jemal A., Bray F. (2021). Global Cancer Statistics 2020: GLOBOCAN Estimates of Incidence and Mortality Worldwide for 36 Cancers in 185 Countries. CA Cancer J. Clin..

[127] Friedman R.J., Rigel D.S., Kopf A.W. (1985). Early Detection of Malignant Melanoma: The Role of Physician Examination and Self-Examination of the Skin. CA Cancer J. Clin..

[128] Magro C.M., Neil Crowson A., Mihm M.C. (2006). Unusual Variants of Malignant Melanoma. Mod. Pathol..

[129] Tang P., Liang Q., Yan X., Xiang S., Sun W., Zhang D., Coppola G. (2019). Efficient Skin Lesion Segmentation Using Separable-Unet with Stochastic Weight Averaging. Comput. Methods Programs Biomed..

[130] MacLellan A.N., Price E.L., Publicover-Brouwer P., Matheson K., Ly T.Y., Pasternak S., Walsh N.M., Gallant C.J., Oakley A., Hull P.R. (2020). The Use of Non-Invasive Imaging Techniques in the Diagnosis of Melanoma: A Prospective Diagnostic Accuracy Study. J. Am. Acad. Dermatol..

[131] Winkler J.K., Sies K., Fink C., Toberer F., Enk A., Abassi M.S., Fuchs T., Haenssle H.A. (2021). Association between Different Scale Bars in Dermoscopic Images and Diagnostic Performance of a Market-Approved Deep Learning Convolutional Neural Network for Melanoma Recognition. Eur. J. Cancer.

[132] Sinclair R., Meah N., Arasu A. (2019). Skin Checks in Primary Care. Aust. J. Gen. Pract..

[133] Rayner J.E., Laino A.M., Nufer K.L., Adams L., Raphael A.P., Menzies S.W., Soyer H.P. (2018). Clinical Perspective of 3D Total Body Photography for Early Detection and Screening of Melanoma. Front. Med..

[134] Primiero C.A., McInerney-Leo A.M., Betz-Stablein B., Whiteman D.C., Gordon L., Caffery L., Aitken J.F., Eakin E., Osborne S., Gray L. (2019). Evaluation of the Efficacy of 3D Total-Body Photography with Sequential Digital Dermoscopy in a High-Risk Melanoma Cohort: Protocol for a Randomised Controlled Trial. BMJ Open.

[135] McClatchey A.I. (2007). Neurofibromatosis. Annu. Rev. Pathol..

[136] Boyd K.P., Korf B.R., Theos A. (2009). Neurofibromatosis Type 1. J. Am. Acad. Dermatol..

[137] Wei C.J., Yan C., Tang Y., Wang W., Gu Y.H., Ren J.Y., Cui X.W., Lian X., Liu J., Wang H.J. (2020). Computed Tomography–Based Differentiation of Benign and Malignant Craniofacial Lesions in Neurofibromatosis Type I Patients: A Machine Learning Approach. Front. Oncol..

[138] Parisi R., Symmons D.P.M., Griffiths C.E.M., Ashcroft D.M. (2013). Global Epidemiology of Psoriasis: A Systematic Review of Incidence and Prevalence. J. Investig. Dermatol..

[139] van de Kerkhof P.C. (1997). the Psoriasis Area and Severity Index and Alternative Approaches for the Assessment of Severity: Persisting Areas of Confusion. Br. J. Dermatol..

[140] Walsh J.A., McFadden M., Woodcock J., Clegg D.O., Helliwell P., Dommasch E., Gelfand J.M., Krueger G.G., Duffin K.C. (2013). Product of the Physician Global Assessment and Body Surface Area: A Simple Static Measure of Psoriasis Severity in a Longitudinal Cohort. J. Am. Acad. Dermatol..

[141] Bozek A., Reich A. (2017). The Reliability of Three Psoriasis Assessment Tools: Psoriasis Area and Severity Index, Body Surface Area and Physician Global Assessment. Adv. Clin. Exp. Med..

[142] Pal A., Chaturvedi A., Garain U., Chandra A., Chatterjee R. Severity Grading of Psoriatic Plaques Using Deep CNN Based Multi-Task Learning. Proceedings of the 2016 23rd International Conference on Pattern Recognition (ICPR).

[143] Pal A., Chaturvedi A., Chandra A., Chatterjee R., Senapati S., Frangi A.F., Garain U. (2022). MICaps: Multi-Instance Capsule Network for Machine Inspection of Munro’s Microabscess. Comput. Biol. Med..

[144] Emam S., Du A.X., Surmanowicz P., Thomsen S.F., Greiner R., Gniadecki R. (2020). Predicting the Long-Term Outcomes of Biologics in Patients with Psoriasis Using Machine Learning. Br. J. Dermatol..

[145] Diepgen T.L., Andersen K.E., Chosidow O., Coenraads P.J., Elsner P., English J., Fartasch M., Gimenez-Arnau A., Nixon R., Sasseville D. (2015). Guidelines for Diagnosis, Prevention and Treatment of Hand Eczema—Short Version. JDDG—J. Ger. Soc. Dermatol..

[146] Garzorz-Stark N., Eyerich K. (2019). Molecular Diagnostics of Hand Eczema. Hautarzt.

[147] Weidinger S., Novak N. (2016). Atopic Dermatitis. Lancet.

[148] Drucker A.M., Wang A.R., Li W.Q., Sevetson E., Block J.K., Qureshi A.A. (2017). The Burden of Atopic Dermatitis: Summary of a Report for the National Eczema Association. J. Investig. Dermatol..

[149] Patella V., Florio G., Palmieri M., Bousquet J., Tonacci A., Giuliano A., Gangemi S. (2020). Atopic Dermatitis Severity during Exposure to Air Pollutants and Weather Changes with an Artificial Neural Network (ANN) Analysis. Pediatr. Allergy Immunol..

[150] Melina A., Dinh N.N., Tafuri B., Schipani G., Nisticò S., Cosentino C., Amato F., Thiboutot D., Cherubini A. (2018). Artificial Intelligence for the Objective Evaluation of Acne Investigator Global Assessment. J. Drugs Dermatol..

[151] Maul L.V., Meienberger N., Kaufmann L. (2020). Role of Artificial Intelligence in Assessing the Extent and Progression of Dermatoses. Hautarzt.

[152] Brewer A.C., Endly D.C., Henley J., Amir M., Sampson B.P., Moreau J.F., Dellavalle R.P. (2013). Mobile Applications in Dermatology. JAMA Dermatol..

[153] De A. (2020). Next-Generation Technologies in Dermatology: Use of Artificial Intelligence and Mobile Applications. Indian J. Dermatol..

[154] Swetter S.M., Tsao H., Bichakjian C.K., Curiel-Lewandrowski C., Elder D.E., Gershenwald J.E., Guild V., Grant-Kels J.M., Halpern A.C., Johnson T.M. (2019). Guidelines of Care for the Management of Primary Cutaneous Melanoma. J. Am. Acad. Dermatol..

[155] Tintle S.M., Keeling J.J., Shawen S.B., Forsberg J.A., Potter B.K. (2010). Traumatic and Trauma-Related Amputations: Part I: General Principles and Lower-Extremity Amputations. J. Bone Jt. Surg. Am..

[156] Tintle S.M., Baechler M.F., Nanos G.P., Forsberg J.A., Potter B.K. (2010). Traumatic and Trauma-Related Amputations: Part II: Upper Extremity and Future Directions. J. Bone Jt. Surg. Am..

[157] Harwell R.C., Ferguson R.L. (1983). Physiologic Tremor and Microsurgery. Microsurgery.

[158] Bodenstedt S., Wagner M., Müller-Stich B.P., Weitz J., Speidel S. (2020). Artificial Intelligence-Assisted Surgery: Potential and Challenges. Visc. Med..

[159] Fagius J., Nordin M., Wall M. (2002). Sympathetic Nerve Activity to Amputated Lower Leg in Humans: Evidence of Altered Skin Vasoconstrictor Discharge. Pain.

[160] Cutrone A., Micera S. (2019). Implantable Neural Interfaces and Wearable Tactile Systems for Bidirectional Neuroprosthetics Systems. Adv. Healthc. Mater..

[161] Parajuli N., Sreenivasan N., Bifulco P., Cesarelli M., Savino S., Niola V., Esposito D., Hamilton T.J., Naik G.R., Gunawardana U. (2019). Real-Time EMG Based Pattern Recognition Control for Hand Prostheses: A Review on Existing Methods, Challenges and Future Implementation. Sensors.

[162] Sohn W., Finley D.S., Jakowatz J., Ornstein D.K. (2010). Robot-Assisted Laparoscopic Transperitoneal Pelvic Lymphadenectomy and Metastasectomy for Melanoma: Initial Report of Two Cases. J. Robot. Surg..

[163] Hyde G.A., Jung N.L., Valle A.A., Bhattacharya S.D., Keel C.E. (2018). Robotic Inguinal Lymph Node Dissection for Melanoma: A Novel Approach to a Complicated Problem. J. Robot. Surg..

[164] Miura J.T., Dossett L.A., Thapa R., Kim Y., Potdar A., Daou H., Sun J., Sarnaik A.A., Zager J.S. (2020). Robotic-Assisted Pelvic Lymphadenectomy for Metastatic Melanoma Results in Durable Oncologic Outcomes. Ann. Surg. Oncol..

[165] Bicknell L.M., Kash N., Kavouspour C., Rashid R.M. (2014). Follicular Unit Extraction Hair Transplant Harvest: A Review of Current Recommendations and Future Considerations. Dermatol. Online J..

[166] Wang X., Shu X., Li Z., Huo W., Zou L., Tang Y., Li L. (2018). Comparison of Two Kinds of Skin Imaging Analysis Software: VISIA(^®^) from Canfield and IPP(^®^) from Media Cybernetics. Ski. Res. Technol..

[167] Holcomb J.D. (2021). Helium Plasma Dermal Resurfacing: VISIA CR Assessment of Facial Spots, Pores, and Wrinkles-Preliminary Findings. J. Cosmet. Dermatol..

[168] Goldsberry A., Hanke C.W., Hanke K.E. (2014). VISIA System: A Possible Tool in the Cosmetic Practice. J. Drugs Dermatol..

[169] Šuchmannová J., Fikrle T., Pizinger K. (2019). Diagnostika Maligního Melanomu s Využitím Celotělového Skenu. Czecho-Slovak Dermatol.

[170] Linming F., Wei H., Anqi L., Yuanyu C., Heng X., Sushmita P., Yiming L., Li L. (2018). Comparison of Two Skin Imaging Analysis Instruments: The VISIA^®^ from Canfield vs. the ANTERA 3D^®^CS from Miravex. Ski. Res. Technol..

[171] Messaraa C., Metois A., Walsh M., Flynn J., Doyle L., Robertson N., Mansfield A., O’Connor C., Mavon A. (2018). Antera 3D Capabilities for Pore Measurements. Ski. Res. Technol..

[172] Messaraa C., Metois A., Walsh M., Hurley S., Doyle L., Mansfield A., O’Connor C., Mavon A. (2018). Wrinkle and Roughness Measurement by the Antera 3D and Its Application for Evaluation of Cosmetic Products. Ski. Res. Technol..

[173] McKoy K., Halpern S., Mutyambizi K. (2021). International Teledermatology Review. Curr. Dermatol. Rep..

[174] Cui Y. (2020). Telemedicine and AI for Dermatology Care in China. Proceedings of the 8th World Congress of Imaging and AI for Skin Diseases.

[175] bei Riesenzellarteriitis D. (2020). Kompass Autoimmun.

[176] Polesie S., Gillstedt M., Kittler H., Lallas A., Tschandl P., Zalaudek I., Paoli J. (2020). Attitudes towards Artificial Intelligence within Dermatology: An International Online Survey. Br. J. Dermatol..

[177] Ching T., Himmelstein D.S., Beaulieu-Jones B.K., Kalinin A.A., Do B.T., Way G.P., Ferrero E., Agapow P.-M., Zietz M., Hoffman M.M. (2018). Opportunities and Obstacles for Deep Learning in Biology and Medicine. J. R. Soc. Interface.

[178] Winkler J.K., Fink C., Toberer F., Enk A., Deinlein T., Hofmann-Wellenhof R., Thomas L., Lallas A., Blum A., Stolz W. (2019). Association between Surgical Skin Markings in Dermoscopic Images and Diagnostic Performance of a Deep Learning Convolutional Neural Network for Melanoma Recognition. JAMA Dermatol..

[179] Haw W.Y.D., Al-janabi A.D., Arents B.W.M.D., Asfour L.D., Exton L.S.D., Grindlay D.D., Khan S.S.D. (2021). Global Guidelines in Dermatology Mapping Project (GUIDEMAP): A Scoping Review of Dermatology Clinical Practice Guidelines. Br. J. Dermatol..

[180] Bera K., Schalper K.A., Rimm D.L., Velcheti V., Haven N. (2019). Artificial Intelligence in Digital Pathology—New Tools for Diagnosis and Precision Oncology. Nat. Rev. Clin. Oncol..

[181] Polesie S., McKee P.H., Gardner J.M., Gillstedt M., Siarov J., Neittaanmäki N., Paoli J. (2020). Attitudes Toward Artificial Intelligence Within Dermatopathology: An International Online Survey. Front. Med..

[182] Nelson C.A., Pérez-Chada L.M., Creadore A., Li S.J., Lo K., Manjaly P., Pournamdari A.B., Tkachenko E., Barbieri J.S., Ko J.M. (2020). Patient Perspectives on the Use of Artificial Intelligence for Skin Cancer Screening: A Qualitative Study. JAMA Dermatol..

[183] Khalid M., Niazi K., Parwani A.V., Gurcan M. (2021). Digital Pathology and Artificial Intelligence. Lancet Oncol..

[184] Steele L., Velazquez-Pimentel D., Thomas B.R. (2021). Do AI Models Recognise Rare, Aggressive Skin Cancers? An Assessment of a Direct-to-Consumer Application in the Diagnosis of Merkel Cell Carcinoma and Amelanotic Melanoma. J. Eur. Acad. Dermatol. Venereol..

[185] Matrix AI Network (2019). Built to Last: Data and Computing Power.

[186] Jaworek-Korjakowska J., Kłeczek P. (2016). Automatic Classification of Specific Melanocytic Lesions Using Artificial Intelligence. BioMed Res. Int..

[187] State Council of China (2017). The Development Plan of the New Generation of Artificial Intelligence.

[188] Liu Y., Jain A., Eng C., Way D.H., Lee K., Bui P., Kanada K., de Oliveira Marinho G., Gallegos J., Gabriele S. (2020). A Deep Learning System for Differential Diagnosis of Skin Diseases. Nat. Med..

[189] Tschandl P., Rinner C., Apalla Z., Argenziano G., Codella N., Halpern A., Janda M., Lallas A., Longo C., Malvehy J. (2020). Human–Computer Collaboration for Skin Cancer Recognition. Nat. Med..

[190] Li X., Yu L., Chen H., Fu C.-W., Xing L., Heng P.-A. (2021). Transformation-Consistent Self-Ensembling Model for Semisupervised Medical Image Segmentation. IEEE Trans. Neural Netw. Learn. Syst..

[191] Pachtrachai K., Vasconcelos F., Chadebecq F., Allan M., Hailes S., Pawar V., Stoyanov D. (2018). Adjoint Transformation Algorithm for Hand-Eye Calibration with Applications in Robotic Assisted Surgery. Ann. Biomed. Eng..

[192] Gao Y., Wang S., Li J., Li A., Liu H., Xing Y. (2017). Modeling and Evaluation of Hand-Eye Coordination of Surgical Robotic System on Task Performance. Int. J. Med. Robot..

[193] Stefano G.B., Kream R.M. (2018). The Micro-Hospital: 5G Telemedicine-Based Care. Med. Sci. Monit. Basic Res..

[194] Spicher N., Schweins M., Thielecke L., Kurner T., Deserno T.M. Feasibility Analysis of Fifth-Generation (5G) Mobile Networks for Transmission of Medical Imaging Data. Proceedings of the 2021 43rd Annual International Conference of the IEEE Engineering in Medicine & Biology Society (EMBC).

[195] Yan X., Ren X. (2021). 5G Edge Computing Enabled Directional Data Collection for Medical Community Electronic Health Records. J. Healthc. Eng..

[196] Psiha M.M., Vlamos P. (2017). IoT Applications with 5G Connectivity in Medical Tourism Sector Management: Third-Party Service Scenarios. Adv. Exp. Med. Biol..

[197] Jell A., Vogel T., Ostler D., Marahrens N., Wilhelm D., Samm N., Eichinger J., Weigel W., Feussner H., Friess H. (2019). 5th-Generation Mobile Communication: Data Highway for Surgery 4.0. Surg. Technol. Int..

[198] Milletari F., Frei J., Aboulatta M., Vivar G., Ahmadi S.-A. (2019). Cloud Deployment of High-Resolution Medical Image Analysis With TOMAAT. IEEE J. Biomed. Health Inform..

[199] Juyal S., Sharma S., Shukla A.S. (2021). Smart Skin Health Monitoring Using AI-Enabled Cloud-Based IoT. Mater. Today Proc..

[200] Juyal S., Sharma S., Harbola A., Shukla A.S. Privacy and Security of IoT Based Skin Monitoring System Using Blockchain Approach. Proceedings of the 2020 IEEE International Conference on Electronics, Computing and Communication Technologies (CONECCT).

[201] Bhadula S., Sharma S. (2020). IoT-Based Skin Monitoring System. Int. J. Recent Technol. Eng..

[202] Juyal S., Sharma S., Shukla A.S. (2021). Security and Privacy Issues in Unified IoT-Based Skin Monitoring System. Mater. Today Proc..

[203] Qian H., Dong B., Yuan J.-J., Yin F., Wang Z., Wang H.-N., Wang H.-S., Tian D., Li W.-H., Zhang B. (2021). Pre-Consultation System Based on the Artificial Intelligence Has a Better Diagnostic Performance Than the Physicians in the Outpatient Department of Pediatrics. Front. Med..

[204] Mao Y., Zhang L. (2021). Optimization of the Medical Service Consultation System Based on the Artificial Intelligence of the Internet of Things. IEEE Access.

[205] Manning C.L. (2019). Artificial Intelligence Could Bring Relevant Guidelines into Every Consultation. BMJ.

[206] Loftus T.J., Tighe P.J., Filiberto A.C., Efron P.A., Brakenridge S.C., Mohr A.M., Rashidi P., Upchurch G.R.J., Bihorac A. (2020). Artificial Intelligence and Surgical Decision-Making. JAMA Surg..

[207] Hinton G.E., Osindero S., Teh Y.-W. (2006). A Fast Learning Algorithm for Deep Belief Nets. Neural Comput..

[208] Talebi-Liasi F., Markowitz O. (2019). Is Artificial Intelligence Going to Replace Dermatologists?. Cutis.

[209] Lucas R.E. (2004). The Industrial Revolution: Past and Future. Annual Report of the Federal Reserve Bank of Minneapolis.

[210] Yampolskiy R.V. (2018). Artificial Intelligence Safety and Security.

[211] Lecun Y., Bengio Y., Hinton G. (2015). Deep Learning. Nature.

[212] Prasad R., Rohokale V. (2020). Artificial Intelligence and Machine Learning in Cyber Security. Cyber Security: The Lifeline of Information and Communication Technology.

[213] Rigby M.J. (2019). Ethical Dimensions of Using Artificial Intelligence in Health Care. AMA J. Ethics.

[214] Gomolin A., Netchiporouk E., Gniadecki R., Litvinov I.V. (2020). Artificial Intelligence Applications in Dermatology: Where Do We Stand?. Front. Med..

[215] O’Sullivan S., Nevejans N., Allen C., Blyth A., Leonard S., Pagallo U., Holzinger K., Holzinger A., Sajid M.I., Ashrafian H. (2019). Legal, Regulatory, and Ethical Frameworks for Development of Standards in Artificial Intelligence (AI) and Autonomous Robotic Surgery. Int. J. Med. Robot. Comput. Assist. Surg..

[216] Dave P., Nambudiri V., Grant-Kels J.M. (2022). The Introduction of “Dr AI”: What Dermatologists Should Consider. J. Am. Acad. Dermatol..

[217] Batbaatar E., Dorjdagva J., Luvsannyam A., Savino M.M., Amenta P. (2017). Determinants of Patient Satisfaction: A Systematic Review. Perspect. Public Health.

[218] Khanna S., Sethi Y., Nambiar A.R. ISkin Specialist—A Big Data Based Expert System for Dermatology. Proceedings of the 2017 IEEE International Conference on Big Data (Big Data).

[219] Muñoz-López C., Ramírez-Cornejo C., Marchetti M.A., Han S.S., Del Barrio-Díaz P., Jaque A., Uribe P., Majerson D., Curi M., Del Puerto C. (2021). Performance of a Deep Neural Network in Teledermatology: A Single-Centre Prospective Diagnostic Study. J. Eur. Acad. Dermatol. Venereol..

[220] Coates S.J., Kvedar J., Granstein R.D. (2015). Teledermatology: From Historical Perspective to Emerging Techniques of the Modern Era: Part I: History, Rationale, and Current Practice. J. Am. Acad. Dermatol..

[221] Yu K.-H., Beam A.L., Kohane I.S. (2018). Artificial Intelligence in Healthcare. Nat. Biomed. Eng..

[222] Nagendran M., Chen Y., Lovejoy C.A., Gordon A.C., Komorowski M., Harvey H., Topol E.J., Ioannidis J.P.A., Collins G.S., Maruthappu M. (2020). Artificial Intelligence versus Clinicians: Systematic Review of Design, Reporting Standards, and Claims of Deep Learning Studies. BMJ.

